# Supplementary material for: Light-melt adhesive based on dynamic carbon frameworks in a columnar liquid-crystal phase
Source: Nat Commun. 2016 Jul 4;7:12094. doi: 10.1038/ncomms12094 (PMC4932191; doi:10.1038/ncomms12094)
Supplement: Supplementary Information — Supplementary Figures 1-30, Supplementary Methods and Supplementary References [file ncomms12094-s1.pdf]

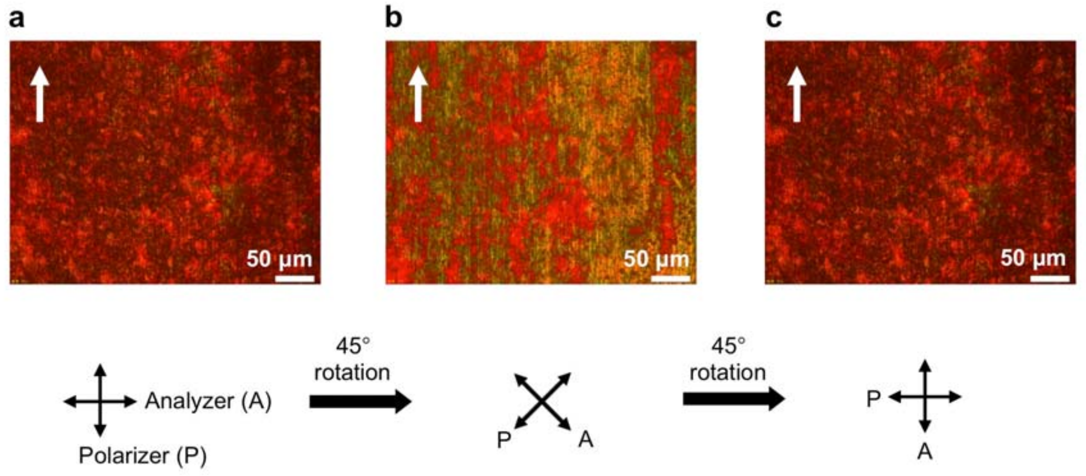

Supplementary Figure 1. POM images of the film of **1** after shearing. The shear was applied in the direction of a white arrow at 100 °C before cooling to room temperature. Double-headed arrows indicate the relative directions of the polarizer and analyzer. **a**, POM image of **1** at room temperature. **b,c,** POM images of **1** upon the clockwise rotation of both polarizer and analyzer by 45° and 90°, respectively.

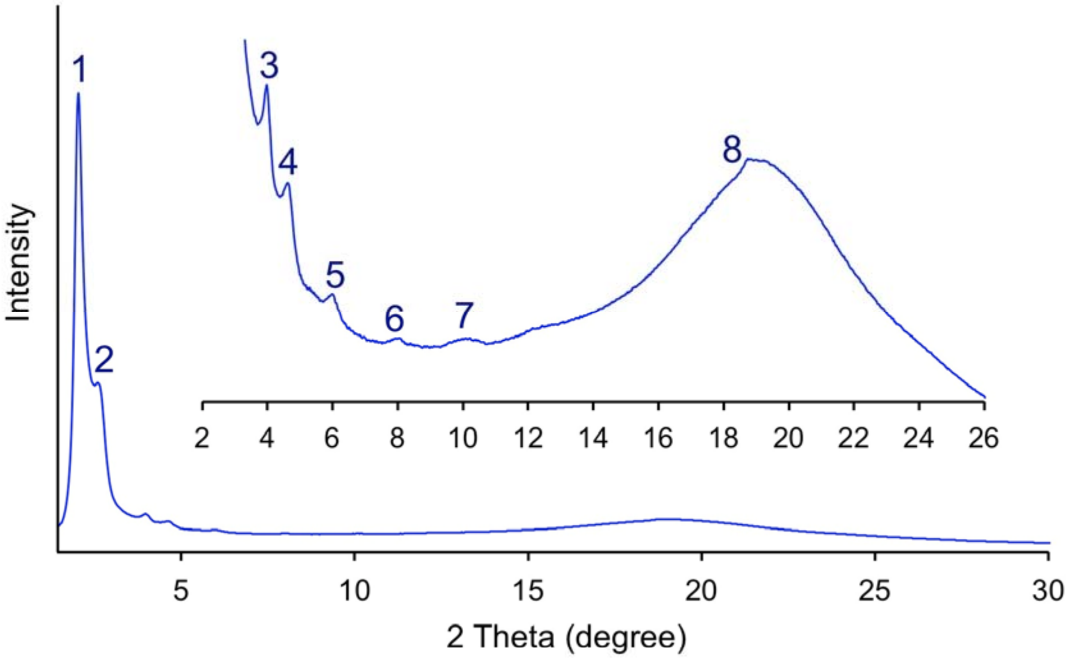

No	2θ /degree	d_{obs} (Å)	d_{calc} (Å)	(hkl)
1	2.04	43.3	44.4	(110)
2	2.60	34.0	standard	(200)
3	3.98	22.2	standard	(220)
4	4.62	19.1	18.8	(130)
5	6.00	14.7	14.8	(330)
6	7.94	11.1	11.1	(440)
7	10.08	8.8	–	–
8	18.86	4.7		(001)

Supplementary Figure 2. XRD pattern of the bulk LC sample of **1** at 100 °C. Inset figure shows the enlarged pattern. Assignments of the observed diffraction peaks were described in the table.

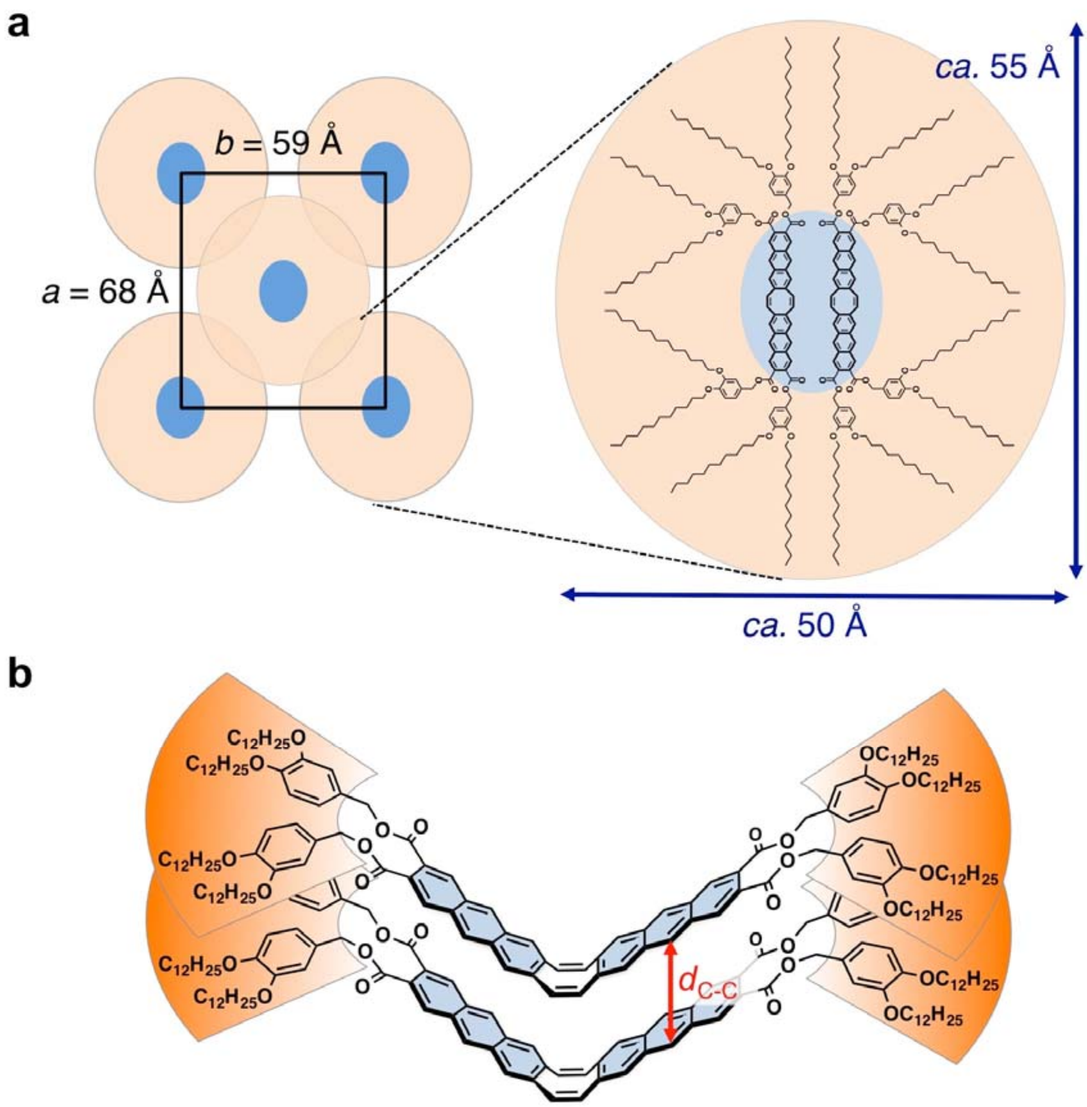

Supplementary Figure 3. Plausible packing structure of **1** in the LC phase. **a**, The repeating cell unit composed of the columnar array structures. **b**, The average interval of the stacking molecules of **1** in the columnar structure, where this interval corresponds to the distance between the reactive carbon sites *d*(C-C) of the anthracene moieties.

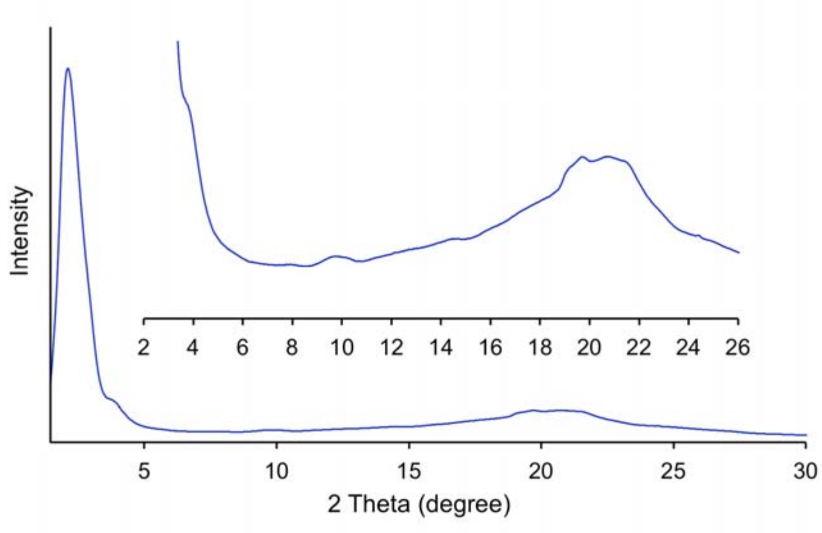

Supplementary Figure 4. XRD pattern of the bulk solid sample of **1** at 25 °C. The bulk sample in the solid phase was prepared upon cooling the liquid crystalline sample at the rate of 5 °C min^{-1}. Inset figure shows the enlarged pattern.

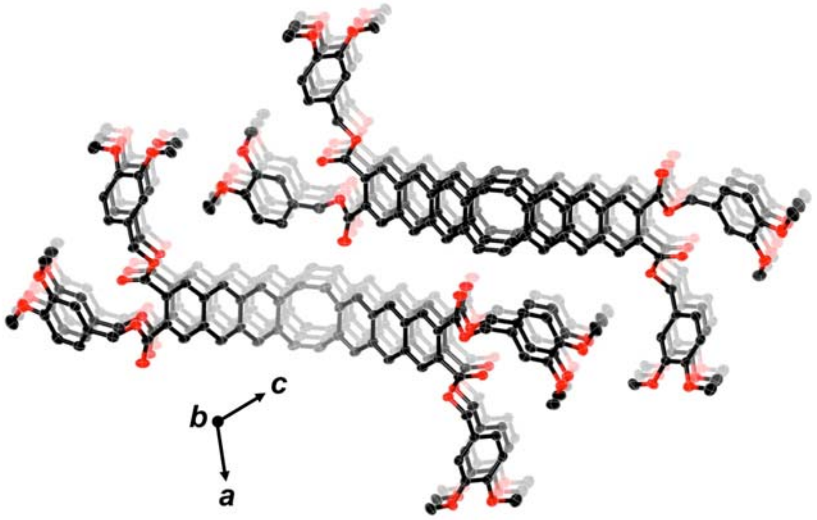

Supplementary Figure 5. X-ray crystal structure of **2**. Hydrogen atoms are omitted for clarity. Thermal ellipsoids are drawn at the 50% probability level.

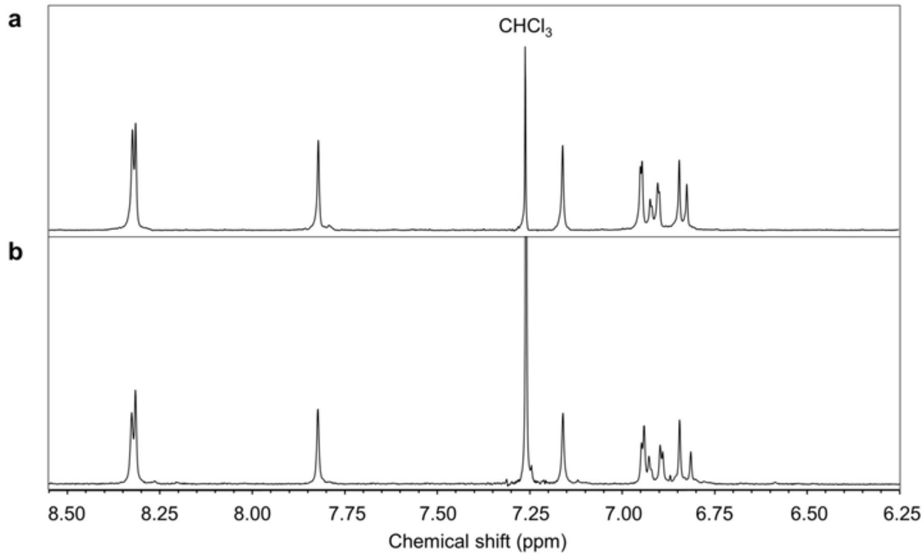

Supplementary Figure 6. ^{1}H NMR analysis on the photoreactivity of the thin film of **1** at 50°C. **a,** Enlarged ^{1}H NMR spectrum of **1** in $CDCl_3$ before photoirradiation. **b,** The thin film was dissolved in $CDCl_3$ after the photoirradiation at 50°C using a hand-held UV lamp (3.2 mW cm^{-2}) for 120 sec. In this condition, significant spectral change was not observed.

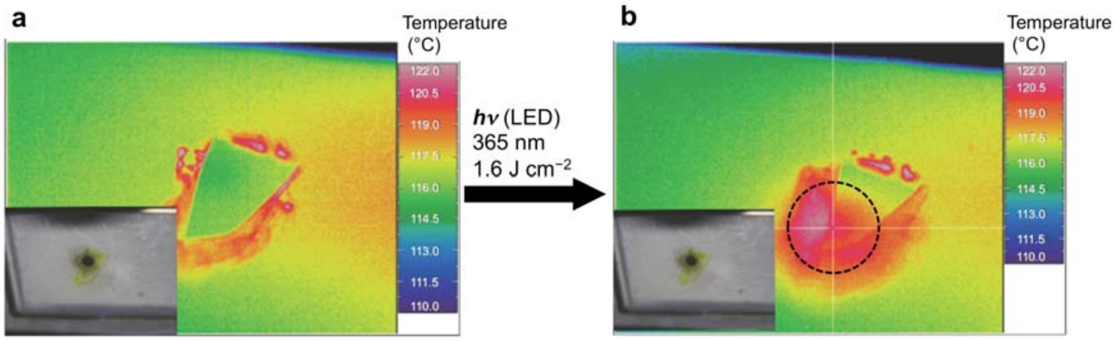

Supplementary Figure 7. Thermography of the LC film of **1**. Thermography before and after the UV-LED irradiation. A dotted-circle indicates the UV irradiated area, where the temperature only increased from 116 °C to 120 °C at a maximum. Inset photographs show the corresponding films.

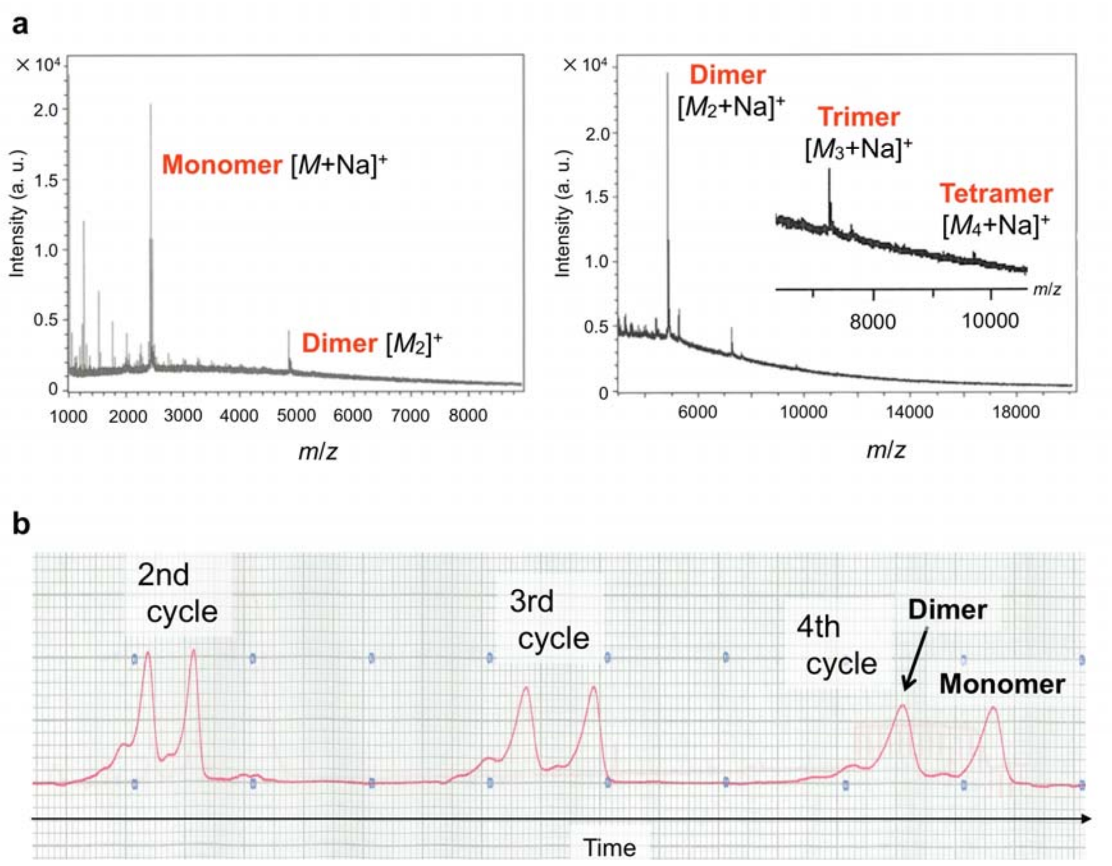

Supplementary Figure 8. Analyses of the photoproducts. **a**, MALDI-TOF MS of the isotropic mixture in the range of *m*/z = 1000–9000 (left) and *m*/z = 3000–20000 (right). Inset figure shows the enlarged spectrum in the range of *m*/z = 7000–10000. Dithranol and NaI were used as a matrix reagent and an additive, respectively. **b**, GPC of the isotropic mixture using $CHCl_3/NEt_3$ (49/1). The photodimer and the unreacted monomer of **1** were isolated with almost same amount of weight.

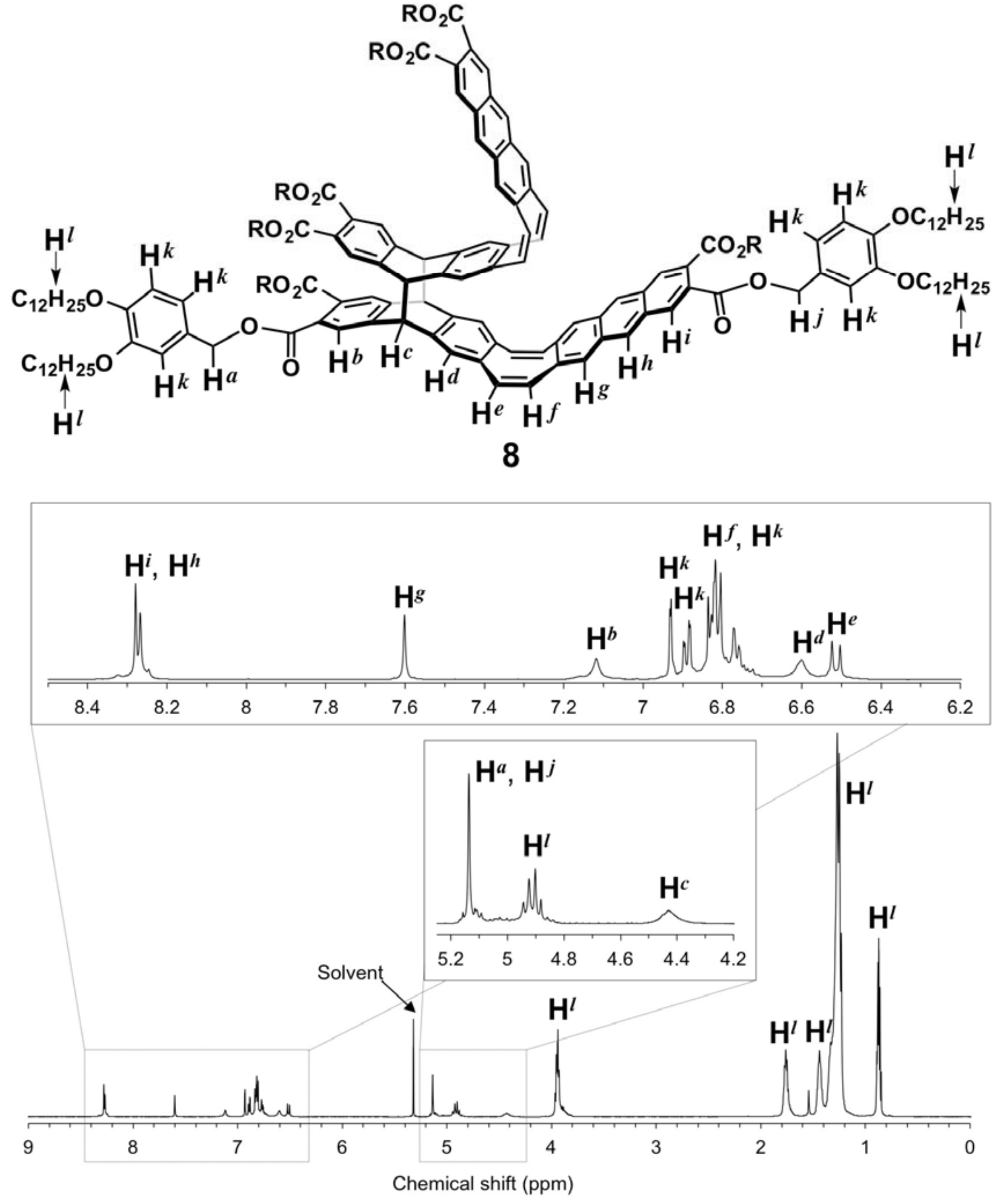

Supplementary Figure 9. ^1H NMR spectrum of the isolated photodimer **8** in CD_2Cl_2.

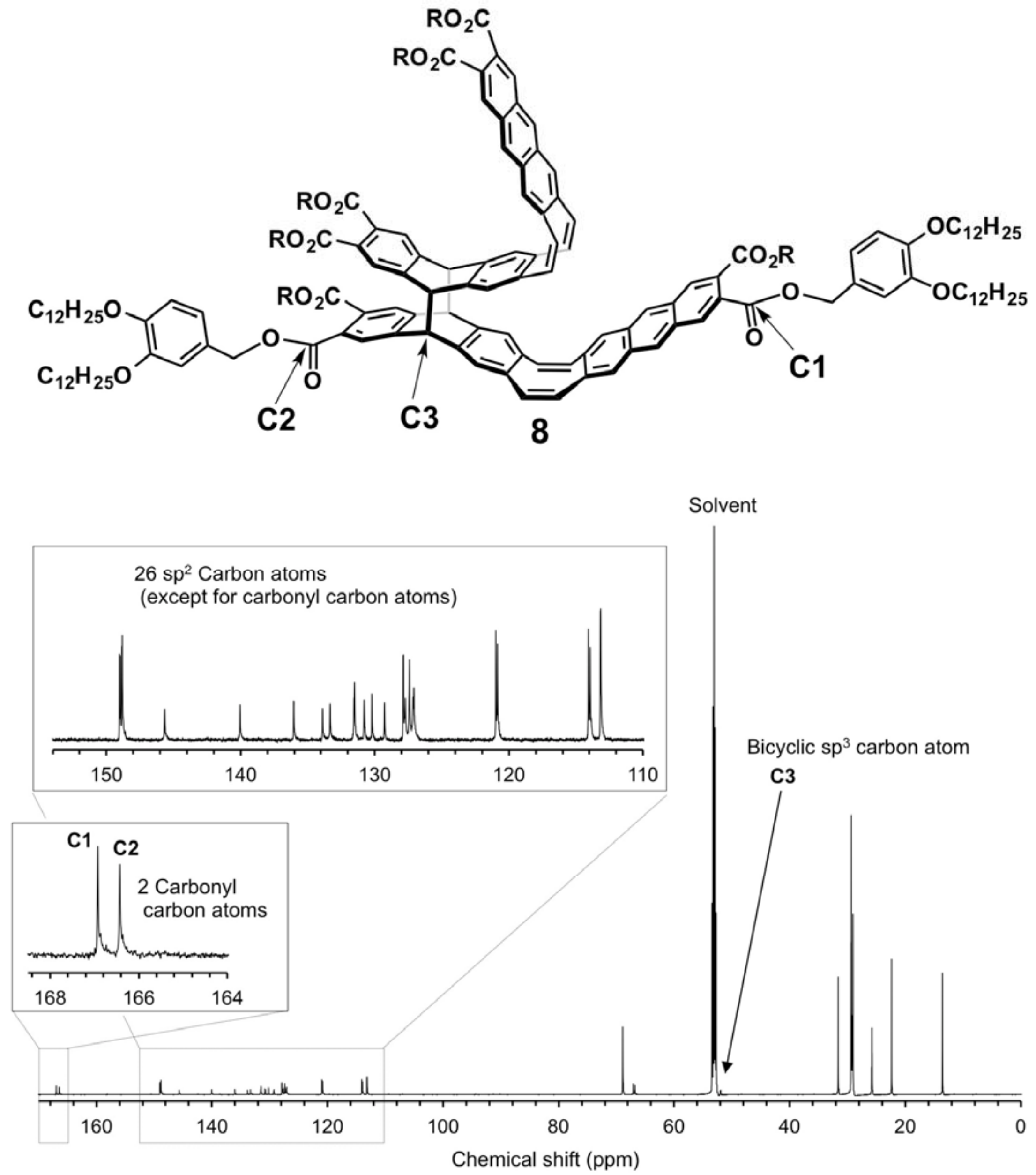

Supplementary Figure 10. ^{13}C NMR spectrum of the isolated photodimer **8** in CD_2Cl_2.

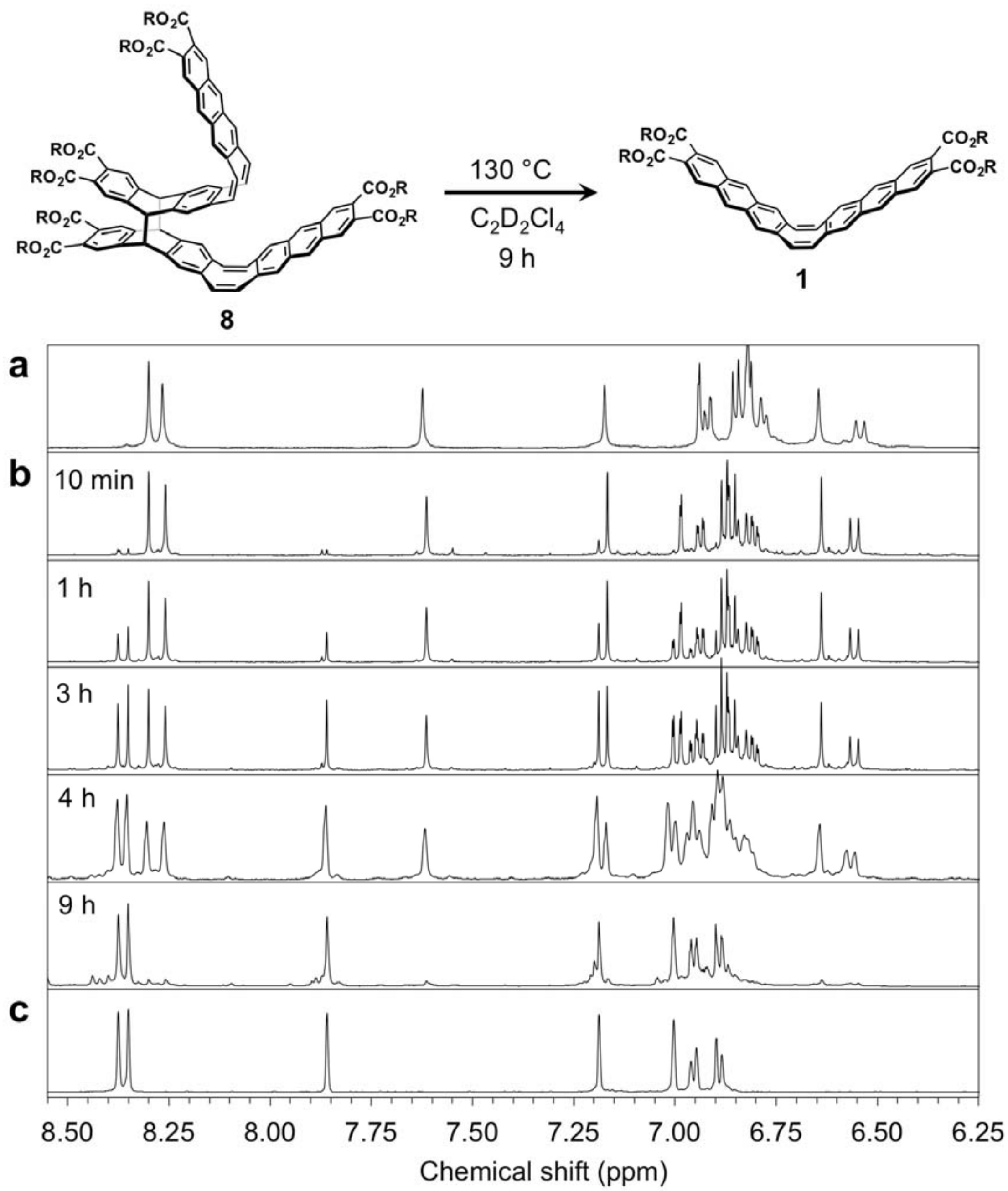

Supplementary Figure 11. Thermal reaction of **8** into **1** in $C_2D_2Cl_4$ solution. **a**, ^{1}H NMR spectrum of **8** at room temperature. **b**, ^{1}H NMR spectra after heating the solution of **8** at 130 °C for 10 min, 1 h, 3 h, 4 h, and 9 h, respectively. **c**, ^{1}H NMR spectrum of the monomer **1** at 130 °C.

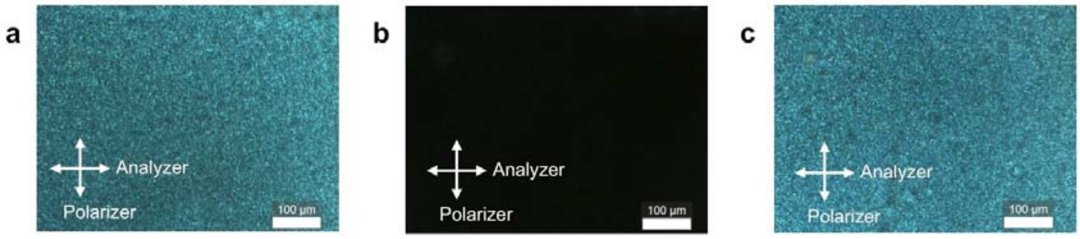

Supplementary Figure 12. Recovery of the bright POM image under the crossed Nicols. **a,** Bright POM image of the LC film of **1** at 100 °C, **b,** Dark POM image of the photoirradiated fluid mixture mainly composed of the unreacted **1** and its photodimer. **c,** The bright POM image was recovered after heating the photoirradiated isotropic mixture above 160°C for 30 min and the following cooling into the LC temperature range (70–135°C) of **1**.

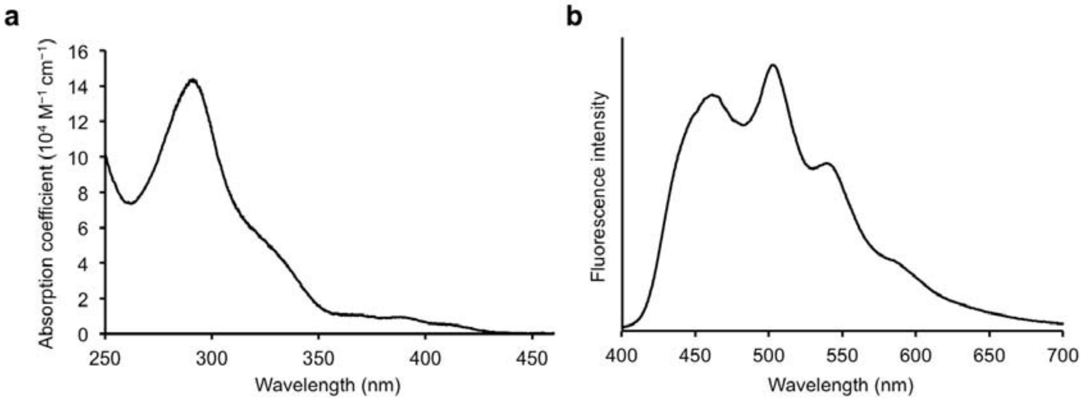

Supplementary Figure 13. Photophysical properties of the isolated photodimer **8**. **a**, UV-visible absorption spectrum in CH_2Cl_2. **b**, Fluorescence spectrum in CH_2Cl_2 with the concentration of [**8**] = 2.4 × 10^{-6} M. Excitation wavelength: 365 nm.

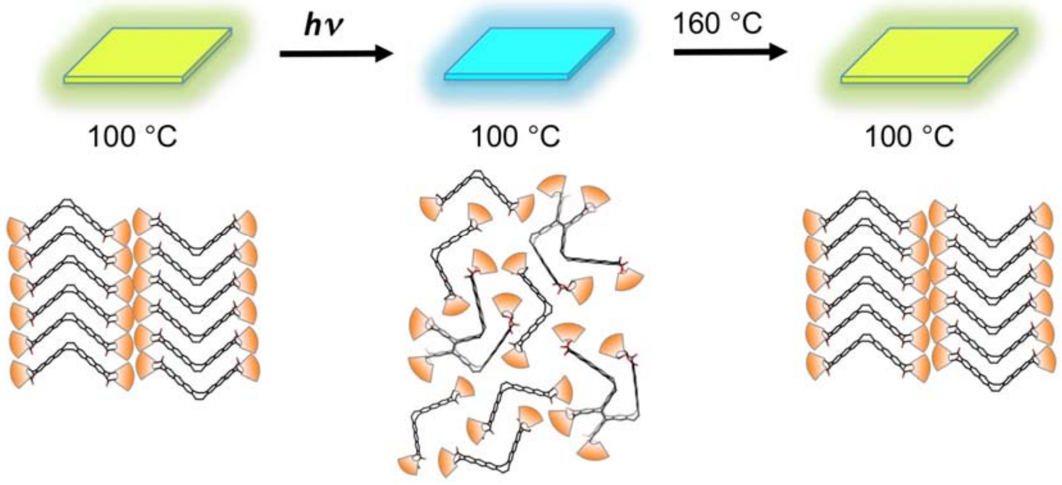

Supplementary Figure 14. Schematic drawings of the fluorescence color change in the film sample of **1** induced by the light-triggered phase transformation and the following thermal restoring reaction.

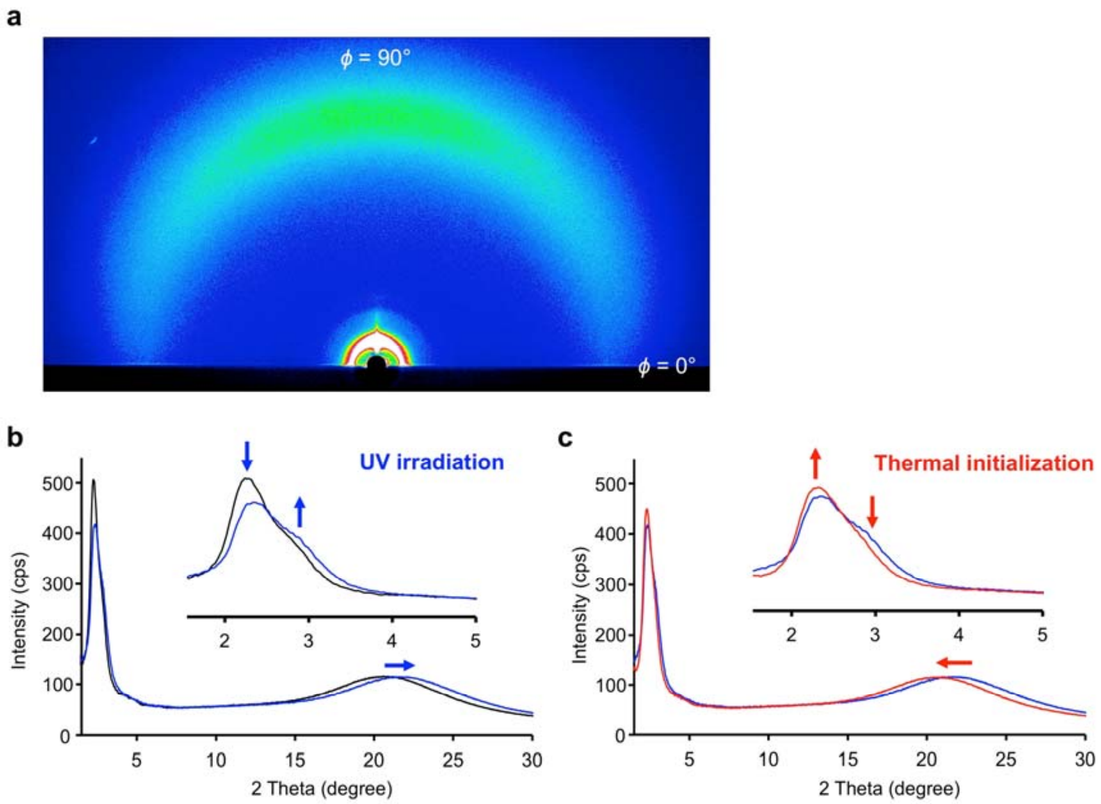

Supplementary Figure 15. GI-XRD patterns of the spin-coated film of **1**. **a**, 2D diffraction image before light irradiation at 100 °C. Diffractions observed in the range of ϕ = 30–60° were integrated to delineate the corresponding 1D intensity profile along the 2θ angle. **b**, The change of the diffraction pattern in the range of 2θ = 1.5 to 30° before (black line) and after light irradiation (blue line) at 100 °C. **c**, The diffraction change before (blue line) and after heating (red line) at 160 °C. Inset figures show the enlarged patterns. All the measurements were performed at 100 °C.

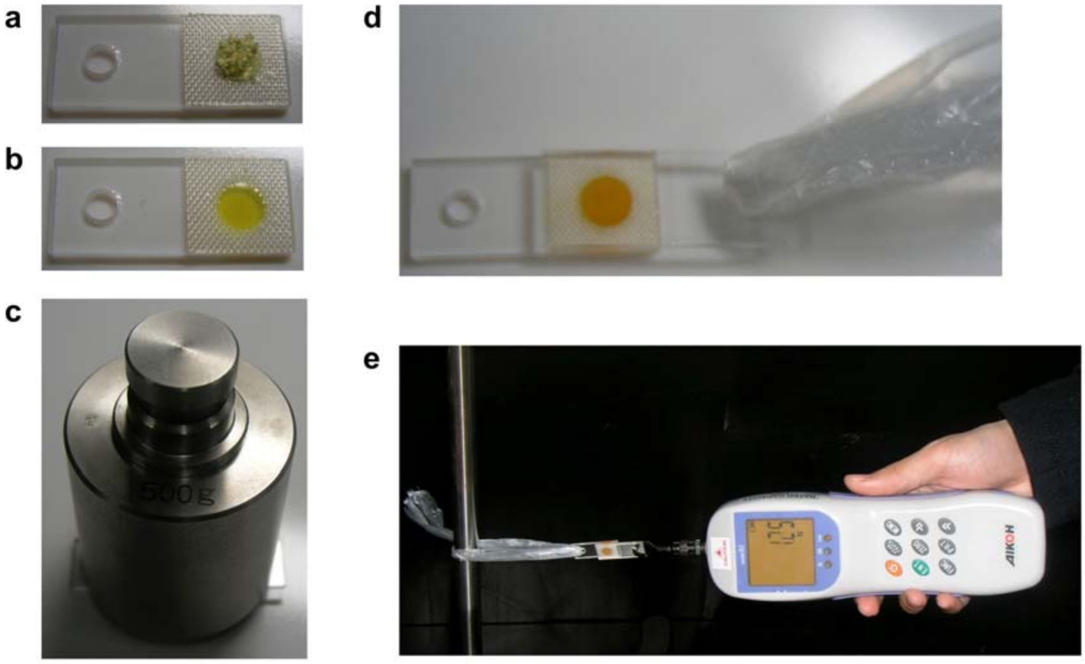

Supplementary Figure 16. Preparation of the film with uniform thickness. **a**, The powder sample of **1** was placed on a glass plate in the hole (6 mmϕ) of a PTFE silicone tape. **b**, The neat liquid sample was formed at 160 °C. **c**, The sample between glass plates was pressed by 0.5 kg weight for 30 min. **d**, The 130-μm-thick film of **1** was prepared between glass plates. **e**, The shear strength was measured using the film sample.

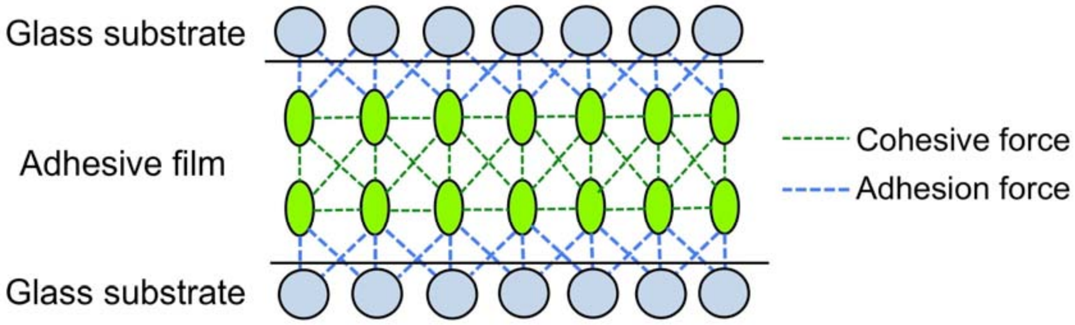

Supplementary Figure 17. Cohesive force and adhesion force.

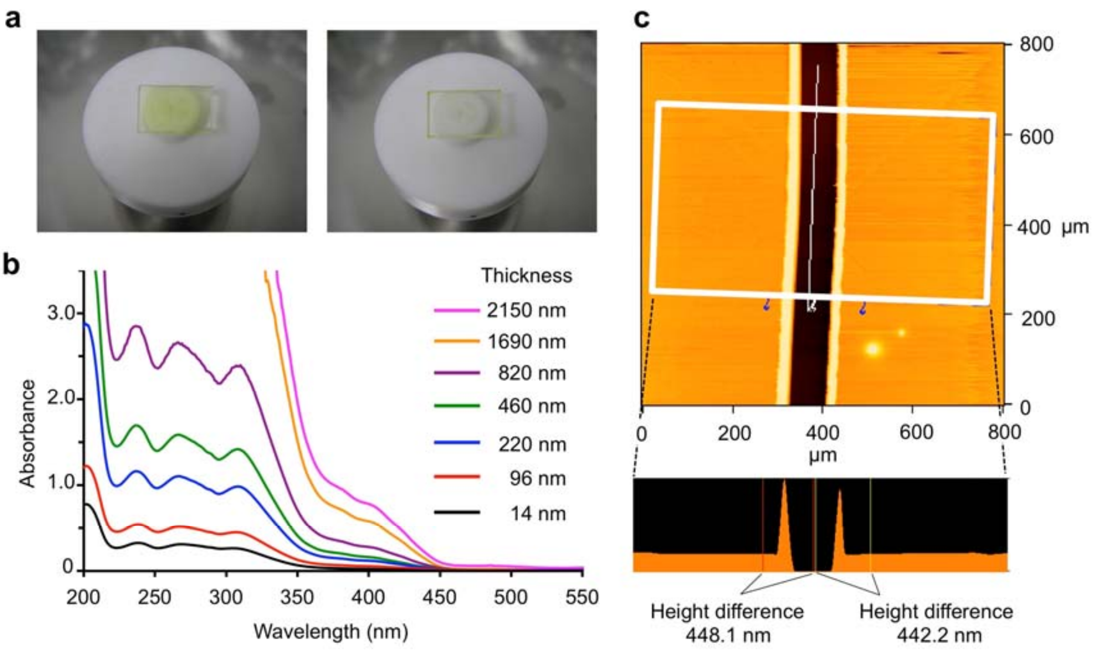

Supplementary Figure 18. Absorption spectra and AFM analysis of the spin-coated films of **1**. **a**, A quartz glass plate on which a $CHCl_3$ solution of **1** was dropped (left), and a thin film prepared on the glass plate after spin-coating (right). **b**, UV-visible absorption spectra of the spin-coated film with different thickness. **c**, Topographic AFM image of the spin-coated film. The bottom figure shows a cross-sectional height profile along the direction perpendicular to the scratched ditch within a white frame of the top image. The film thickness was determined by the measurement of the height difference.

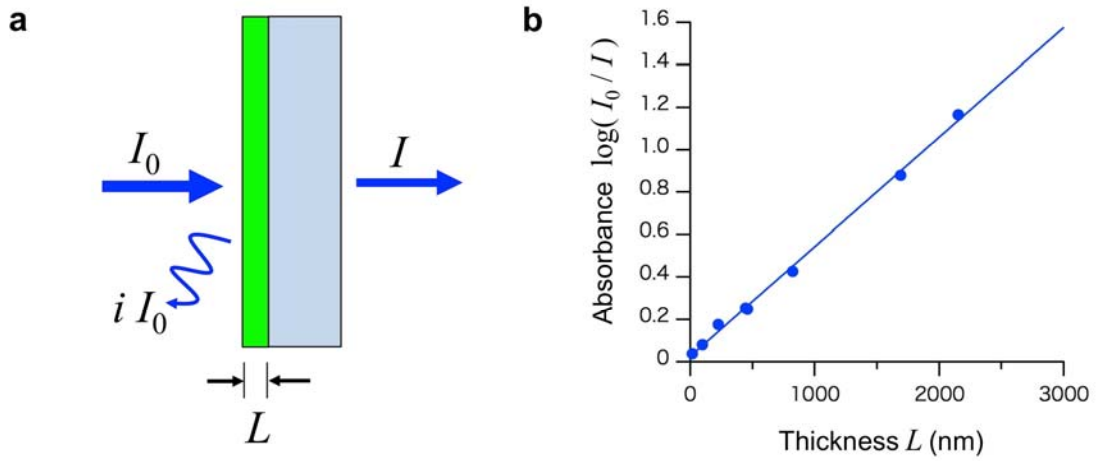

Supplementary Figure 19. Light transmittance of the spin-coated films of **1**. **a**, The transmittance of the 365-nm UV light through the thin film of **1** (green) prepared on a quartz glass plate (blue). **b**, Linear fitting in the plot of the absorbance against the film thickness.

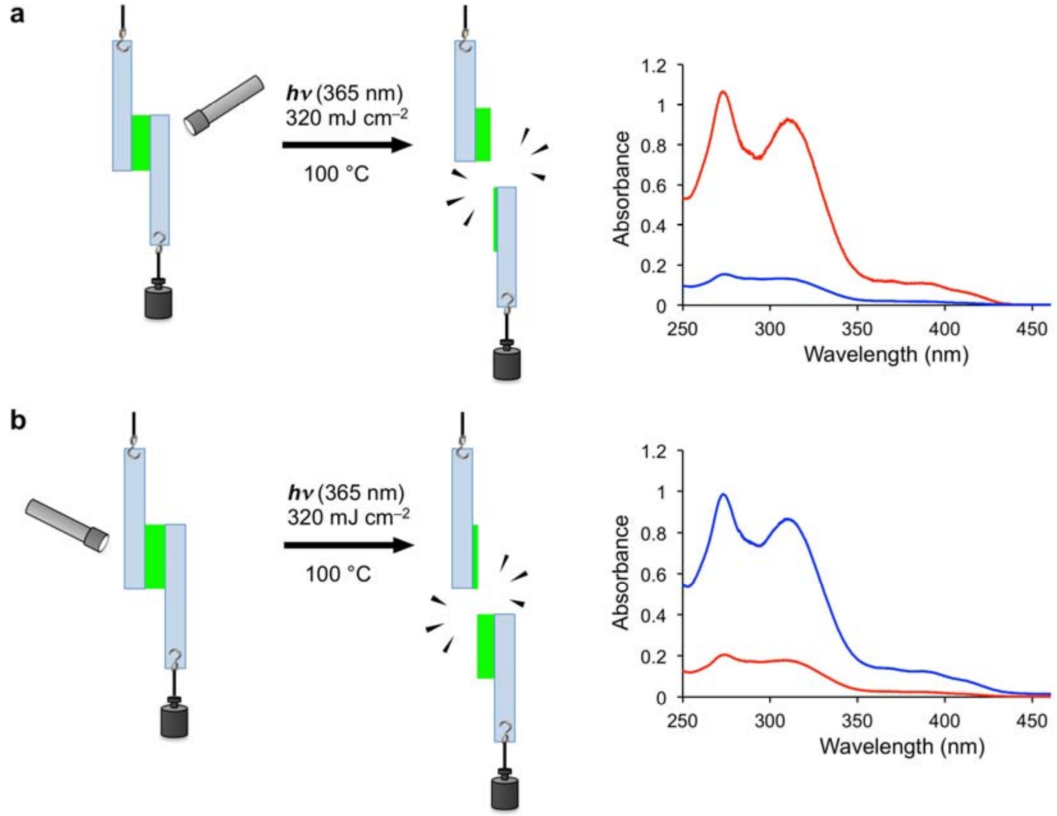

Supplementary Figure 20. Photoinduced detachment near the interface. **a**,**b**, Light irradiation on a 20-μm-thick film of **1** (green) from different directions (left). Relative absorption spectra of the residue solutions (right). The blue spectra correspond to the residue adhering to the dropped glass plate, while the red spectra correspond to the residue adhering to the hanged glass plate.

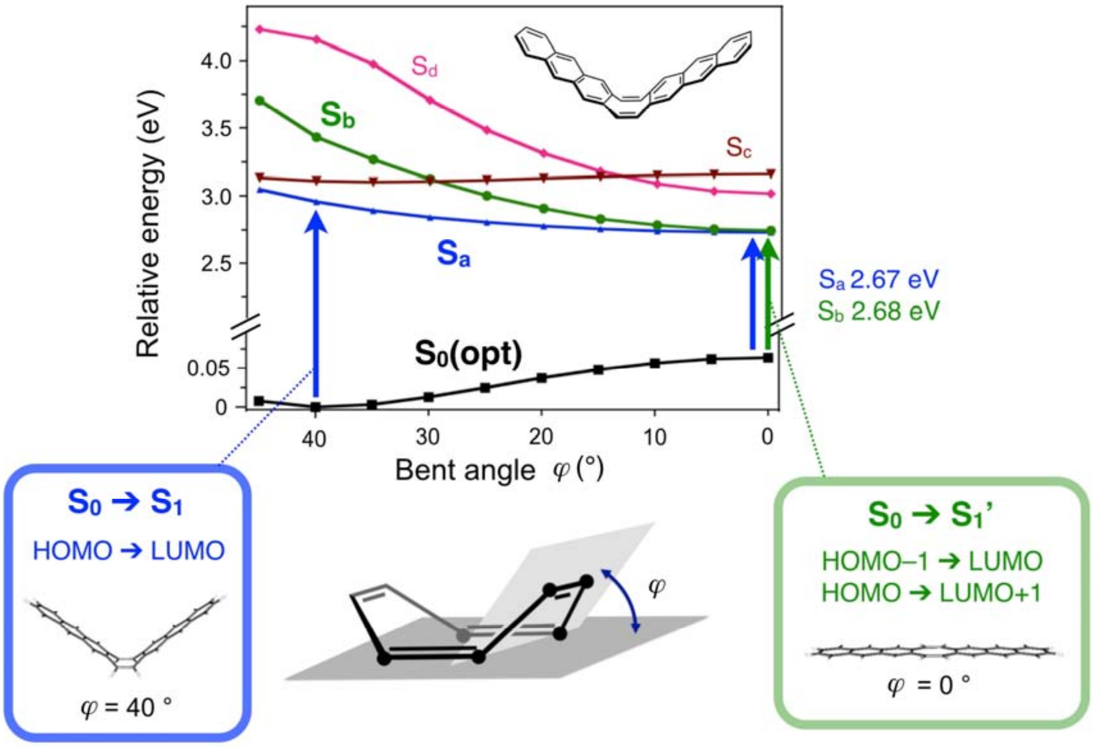

Supplementary Figure 21. Calculated energy diagram for the carbon framework **3**. Energy levels of the ground state S_0 and excited states S_a, S_b, S_c, and S_d for **3** with fixed bent angles φ of the 8-membered ring at the center of the molecular structure. The DFT and TD-DFT calculations were performed at the PBE0/def-SV(P) and TD-PBE0/def-SV(P) levels of theory.

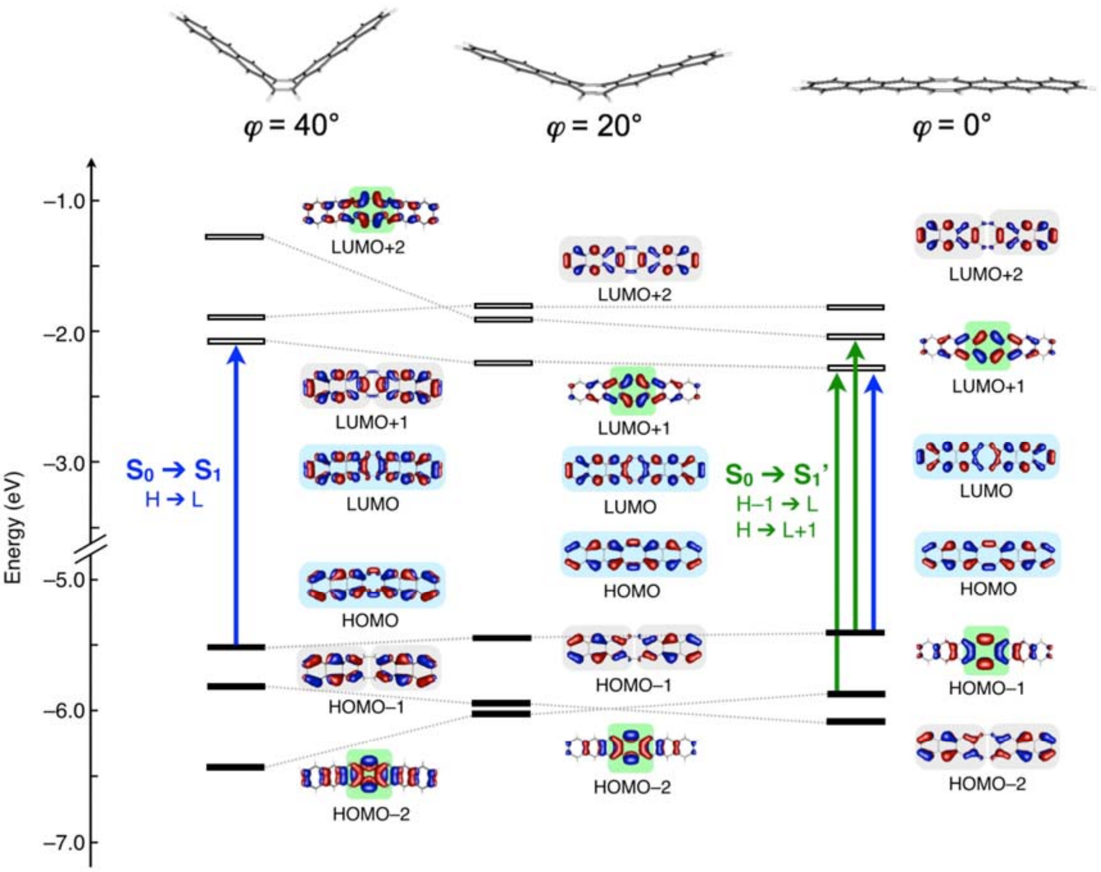

Supplementary Figure 22. Molecular orbital analysis of the carbon framework **3**. Kohn-Sham molecular orbitals and their energy levels of **3** with different bent angles. The constrained DFT geometry optimizations in the ground state S_0 and the TD-DFT calculations were performed at the PBE0/def-SV(P) and TD-PBE0/def-SV(P) levels of theory.

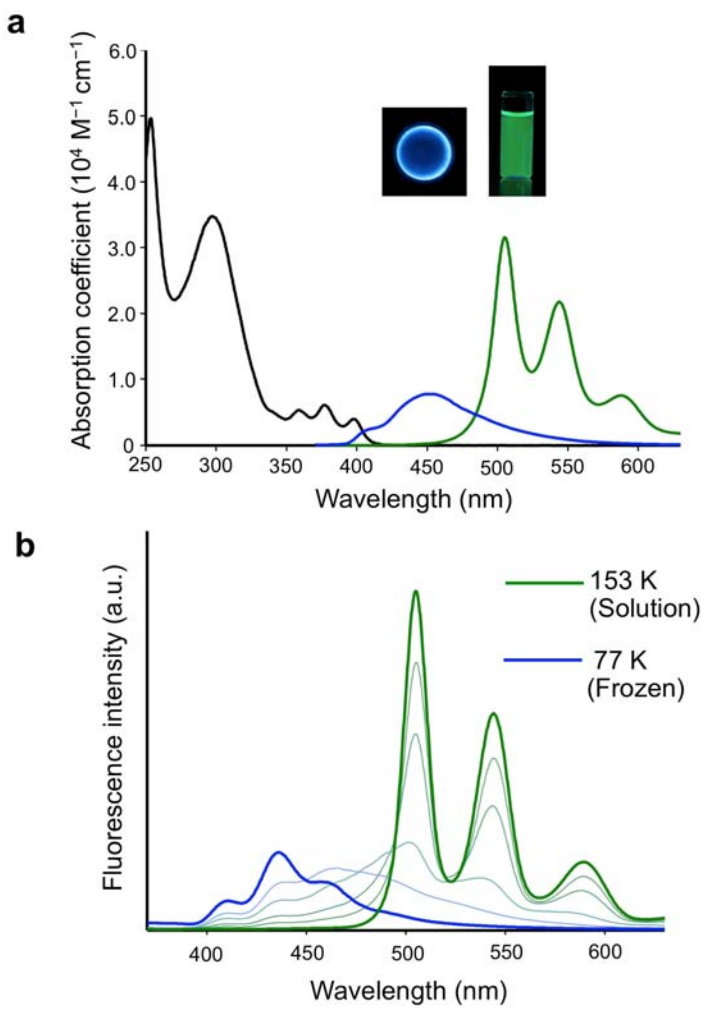

Supplementary Figure 23. Photophysical properties of **3**. **a**, UV-visible absorption (black) and fluorescence (green, [**3**] = 2.0 × 10^{-5} M) spectra of **3** in CH_2Cl_2. Blue line shows a fluorescence spectrum of a polymethyl methacrylate (PMMA) matrix containing **3** in a 1.0 wt% concentration. Excitation wavelength: 365 nm. Inset figures show the photographs of the PMMA matrix (left) and the CH_2Cl_2 solution (right). **b**, Viscosity-dependent fluorescence spectra of **3** in 2-methyltetrahydrofuran (MTHF) from 153 K (solution, green) to 77 K (frozen glass, blue). [**3**] = 1.0 × 10^{-5} M. Excitation wavelength: 330 nm. Melting point of MTHF: 137 K.

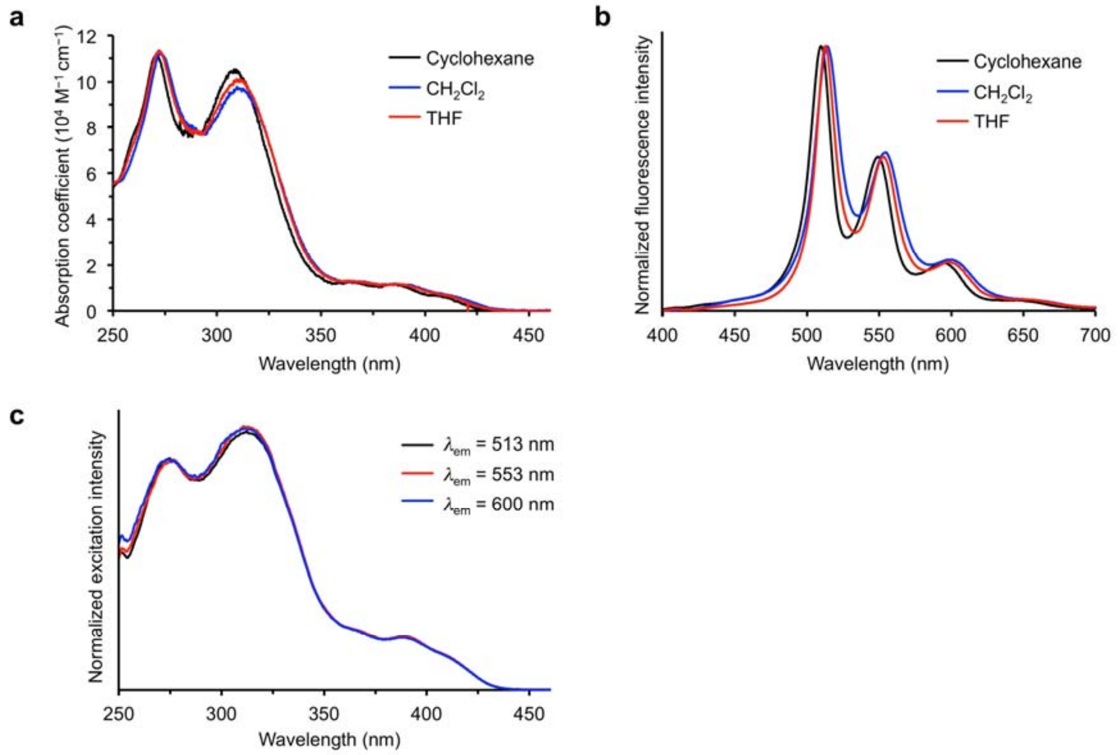

Supplementary Figure 24. Photophysical properties of **1**. **a**, UV-visible absorption spectra in various solvents. **b**, Fluorescence spectra in various solvents with the concentration of [**1**] = 3.2 × 10^{-6} M. Excitation wavelength: 365 nm. In THF, for example, the fluorescence band showed the maximum intensity at 513 nm, accompanied by the vibronic bands at 553 nm and 600 nm. **c**, Excitation spectra in THF at different emission wavelengths λ_{em}.

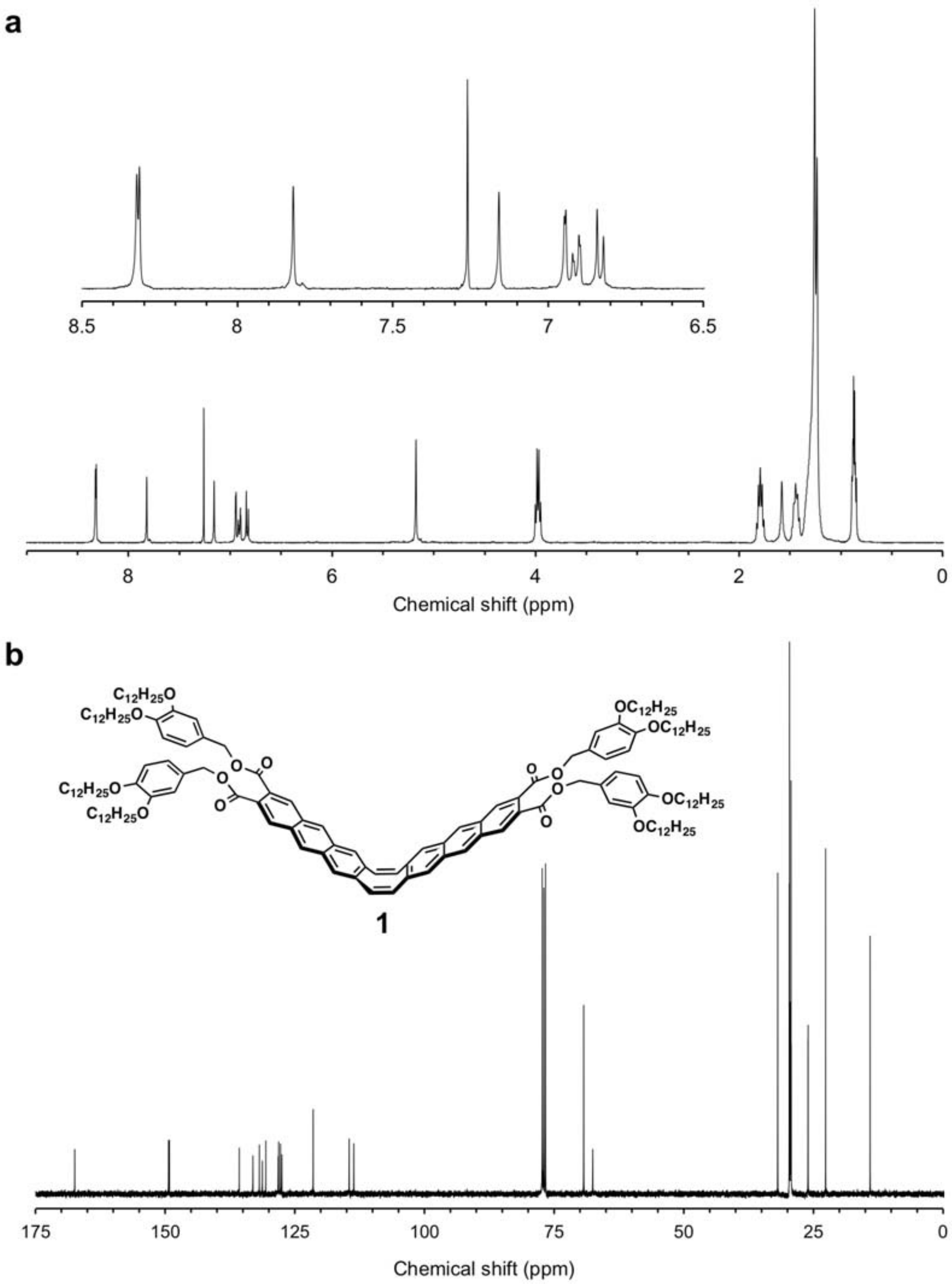

Supplementary Figure 25. a, ^{1}H NMR and **b,** ^{13}C NMR spectra of **1** in $CDCl_3$.

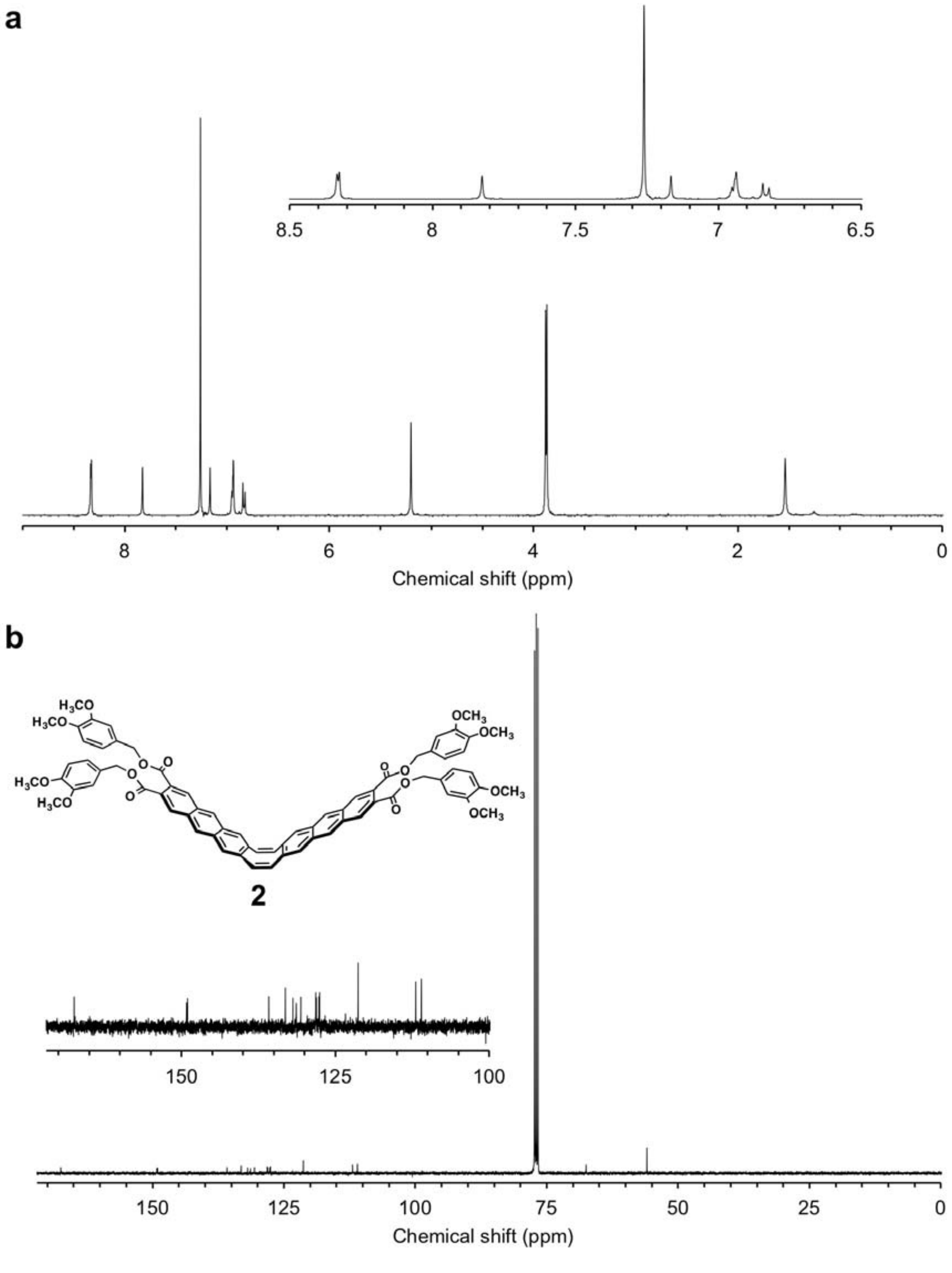

Supplementary Figure 26. a, ^{1}H NMR and **b,** ^{13}C NMR spectra of **2** in $CDCl_3$.

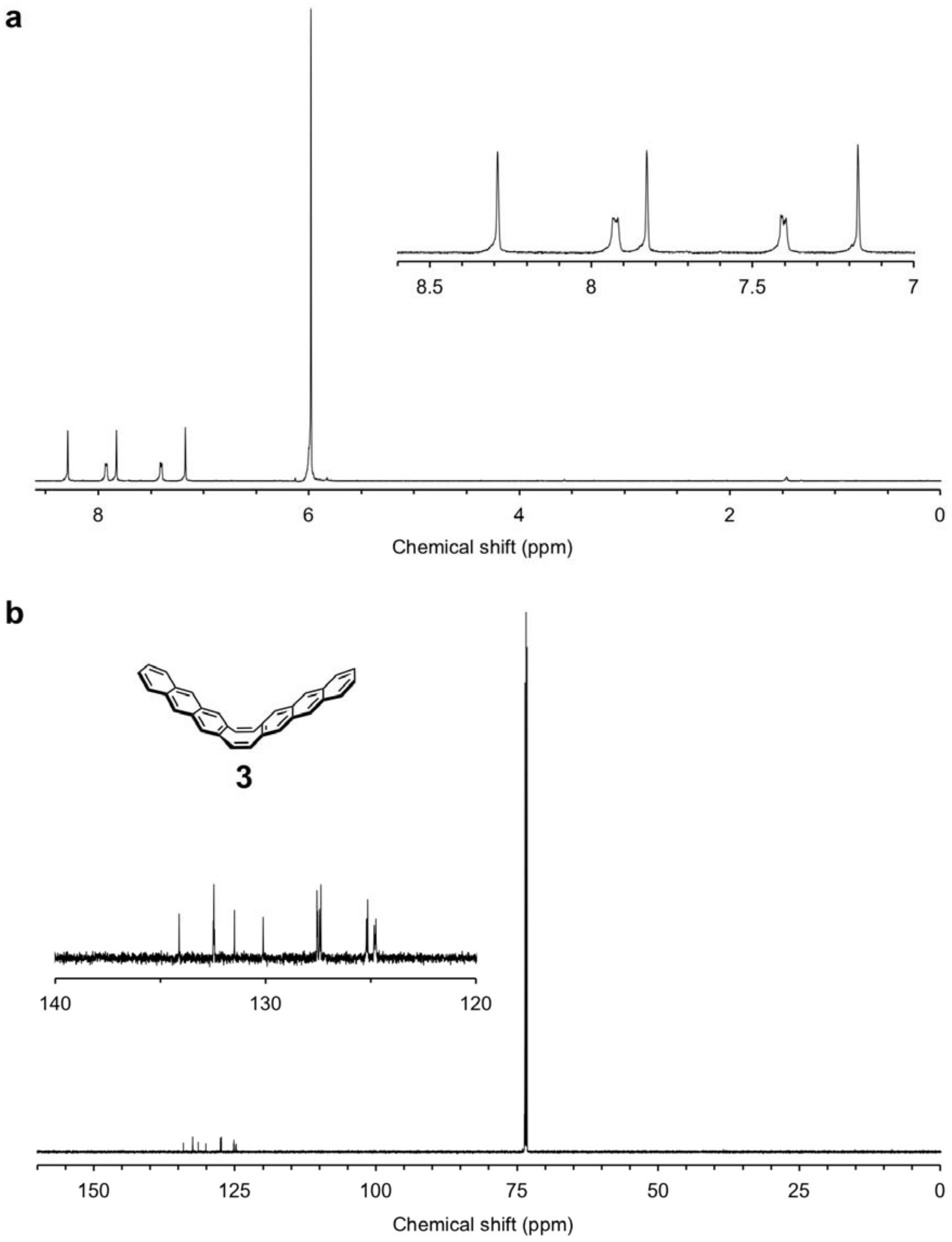

Supplementary Figure 27. a, ^{1}H NMR and **b,** ^{13}C NMR spectra of **3** in $C_2D_2Cl_4$ at 100 °C.

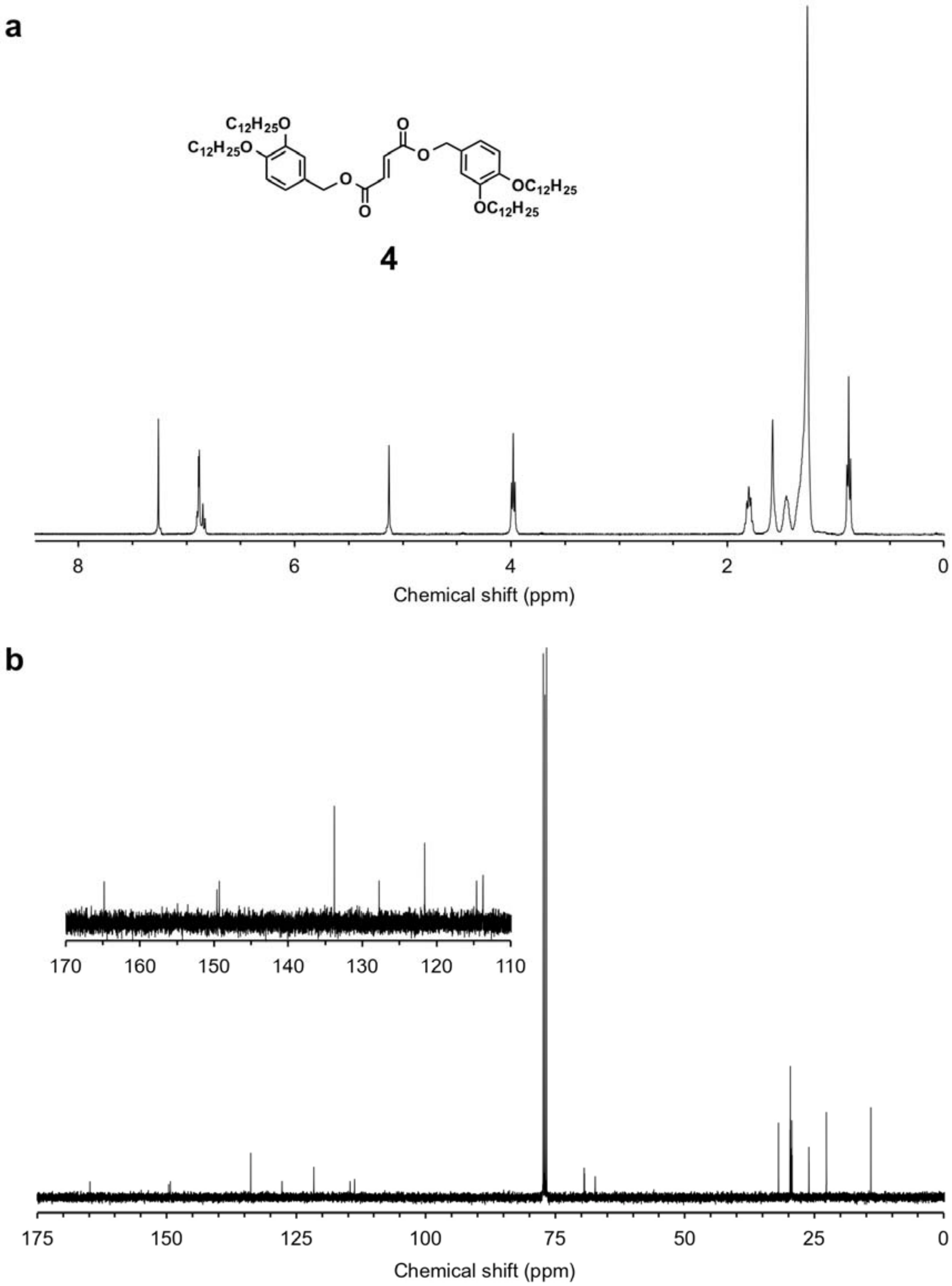

Supplementary Figure 28. a, ^{1}H NMR and **b,** ^{13}C NMR spectra of **4** in $CDCl_3$.

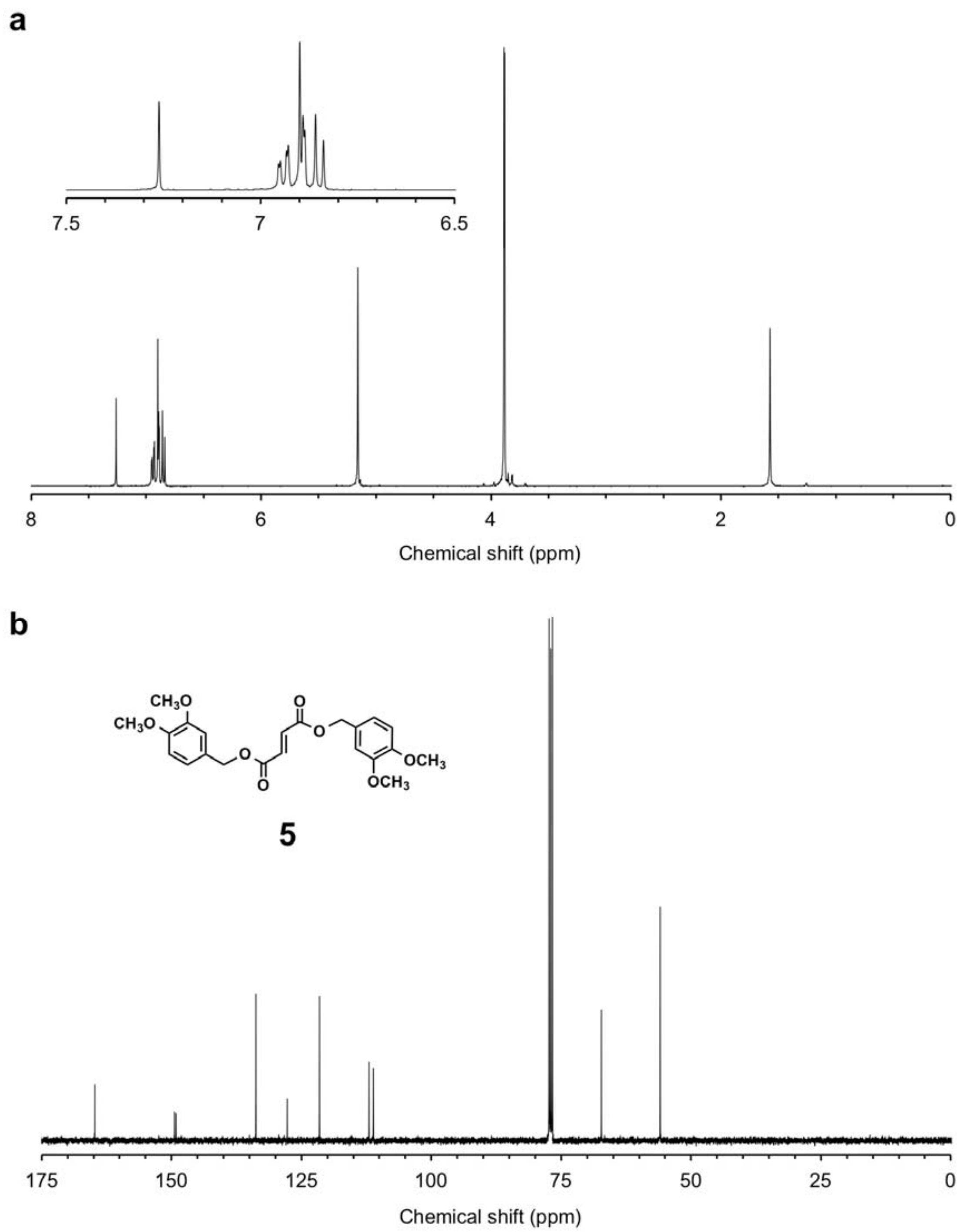

Supplementary Figure 29. a, ^{1}H NMR and **b,** ^{13}C NMR spectra of **5** in $CDCl_3$.

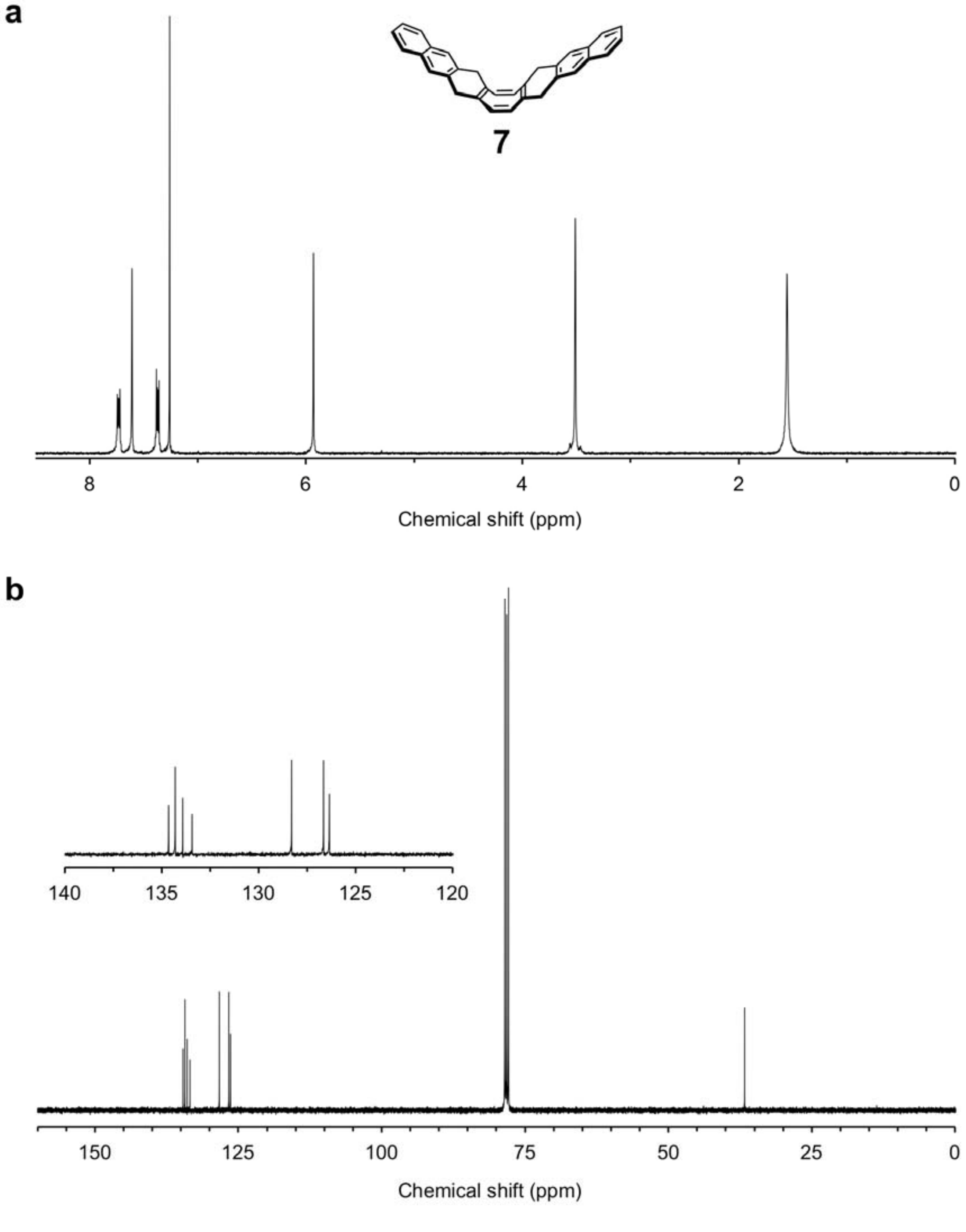

Supplementary Figure 30. a, ^{1}H NMR and **b,** ^{13}C NMR spectra of **7** in $CDCl_3$.

Supplementary Methods

All reagents and solvents were obtained from commercial suppliers and used as received without further purification unless otherwise stated. Tetrahydrofuran (THF) and CH_2Cl_2 were dried using Glass Contour solvent purification system. Polymethyl methacrylate (PMMA) has a weight-average molecular weight (M_w) of 15,000 with a glass transition temperature (T_g) of 105 °C. Polyvinyl alcohol (PVA) has a number-average degree of polymerization of 2000 with a degree of saponification of 0.8. Thin layer chromatography (TLC) was performed on the glass plates coated with 0.25 mm thickness of Silica Gel 60 F-254 (Merck). Column chromatography was performed using neutral silica gel PSQ60B or PSQ100B (Fuji Silysia Chemical). Recycling preparative gel permeation chromatography (GPC) was performed using LC-918 (Japan Analytical Industry) equipped with polystyrene gel columns JAIGEL 1H and 2H, or 2H and 2.5H (Japan Analytical Industry). High performance liquid chromatography (HPLC) was performed using JAI LC-918 (Japan Analytical Industry) equipped with silica gel column (Wakopak Wakosil-II 5SIL Prep). Melting points were determined with a Yanaco MP-S3 instrument. ^{1}H and ^{13}C NMR spectra were recorded on a JEOL AL-400 (400 MHz for ^{1}H, and 100 MHz for ^{13}C NMR) spectrometer or a JEOL JNM-A600 spectrometer equipped with UltraCOOL probe (600 MHz for ^{1}H, and 150 MHz for ^{13}C NMR). The NMR spectra were measured in $CDCl_3$, CD_2Cl_2 or $C_2D_2Cl_4$ (1,1,2,2-tetrachloroethane-d_2) at room temperature unless otherwise stated. Chemical shifts were referenced to the residual solvent protons in the ^{1}H NMR (7.26 ppm in $CDCl_3$, 5.32 ppm in CD_2Cl_2, and 5.98 ppm in $C_2D_2Cl_4$) and the solvent carbons in the ^{13}C NMR (77.0 ppm in $CDCl_3$, 53.1 ppm in CD_2Cl_2, and 73.7 ppm in $C_2D_2Cl_4$). Mass spectrometry (MS) was recorded on a Bruker micrOTOF Focus using the atmospheric pressure chemical ionization (APCI) time of flight (TOF) method in the positive-ion mode in toluene and on a Bruker ultraflex III using the matrix-assisted laser desorption/ionization (MALDI) TOF method. Single crystal X-ray diffraction (XRD) measurement was performed on a Rigaku X-ray diffractometer equipped with a molybdenum MicroMax-007 HF rotating anode X-ray generator (Mo Kα, 0.7107 Å), VariMax-Mo optics, and a Saturn 70 CCD detector. XRD measurements of bulk liquid crystal (LC) samples using a transmission method were

performed on a Rigaku X-ray diffractometer equipped with a copper FR-E rotating anode X-ray generator (Cu Kα, 1.5418 Å), VariMax-Cu optics, and an R-AXIS IV 2D imaging plate detector. The bulk sample was packed in a Lindemann glass capillary (Hilgenberg GmbH) with a diameter of 1.0 mm. Grazing incidence (GI) XRD measurement of a thin film was performed using the same X-ray diffractometer equipped with a hot stage accessory. Ozone treatment of the thin film was performed on a vacuum excimer UV chamber system H0017 (USHIO). Differential scanning calorimeter (DSC) was performed on an SII Exstar 6000 DSC 6200, calibrated using an Al_2O_3 standard sample in a sealed Al pan. Polarized optical microscopy (POM) was performed on a Leica DM2500 P. Thickness of more than 1 μm-thick film was measured using a digital micrometer QuantuMike Series 293 (Mitutoyo). Ultimate shear strength was measured with a push-pull type digital force gauge RZ-10 (AIKOH engineering). Tempax borosilicate glass plates (SANRITSU, 2.0 mm thickness) were used for sandwiching the adhesive material. Preparation of uniform 130-μm-thick films was conducted using a polytetrafluoroethylene (PTFE) silicone tape AGF-100 FR (CHUKOH) as a spacer. Temperature of the sample was controlled by a hot plate CHP-170DN (AS ONE). Accurate temperature on the hot plate was monitored using an infrared thermometer IT-545 (HORIBA). Quartz glass plates (Hiraoka Special Glass, 1.0 mm thickness) were used for the evaluation of the light transmittance through spin-coated films. These thin films with less than 1 μm thickness were prepared using a spin coater 1H-D7 (MIKASA), whose thickness was determined with an atomic force microscope (AFM), SII Nanopics 2100. As a light source, a UV-400 series UV-LED (Keyence, UV-50H type, 365 nm) equipped with a UV-L3 lens unit or a hand-held UV lamp LUV-16 (AS ONE, 365 nm) was used. Irradiance from these light sources on the sample was measured using an ultraviolet irradiance meter UIT-150 (USHIO). Thermography was performed using TH9100WR (NEC Avio Infrared Technologies). UV-visible absorption spectra of solution and spin-coated films were recorded on Shimadzu UV-3150 and Agilent 8453 spectrometers, respectively. Fluorescence spectra in solution were recorded on a Hitachi F-4500 spectrometer. Variable-temperature fluorescence spectra were recorded on a JASCO FP-6500 spectrophotometer equipped with HPC-503 high temperature cell for film samples.

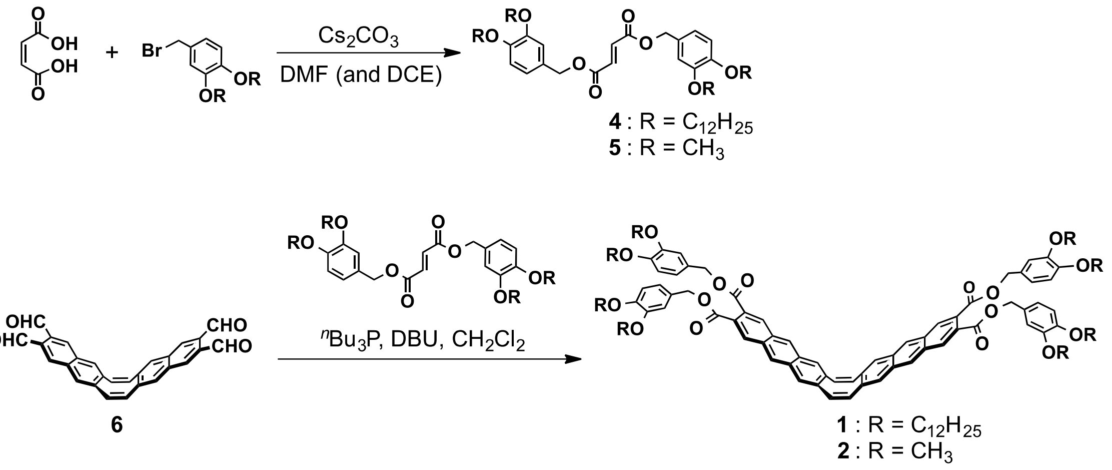

Compounds **1** and **2** were synthesized using an acene elongation reaction[1]. The precursors, 3,4-bis(dodecyloxy)-benzyl bromide and compound **6** were synthesized according to the literature procedures[2,3].

Synthesis of 4. To a solution of maleic acid (81.7 mg, 0.70 mmol) in dimethylformamide (DMF, 3 mL) was added Cs_2CO_3 (688 mg, 2.11 mmol), and the reaction mixture was stirred at room temperature for 50 min. A solution of 3,4-bis(dodecyloxy)benzyl bromide (960 mg, 1.78 mmol) in 1,2-dichloroethane (DCE, 3 mL) was added dropwise, and the reaction mixture was stirred at room temperature for 1 day. After that, 3,4-Bis(dodecyloxy)benzyl bromide (960 mg, 1.78 mmol) was further added to the mixture and then stirred at 60 °C for 2 days. After cooling to room temperature, the reaction was quenched with water and the reaction mixture was extracted with CH_2Cl_2. The extract was washed with brine and dried over anhydrous Na_2SO_4. After removal of the solvent under reduced pressure, the residue was purified by column chromatography over silica gel using CH_2Cl_2/hexane (2/1) to afford bis[3,4-bis(dodecyloxy)benzyl] fumarate (**4**) (305 mg, 0.30 mmol, 43%) as a white solid.

mp: 85.0–86.0 °C; ^{1}H NMR (400 MHz, $CDCl_3$) δ (ppm) 6.90–6.88 (m, 6H), 6.40 (d, J = 8.0 Hz, 2H), 5.13 (s, 4H), 3.98 (t, J = 6.8 Hz, 8H), 1.84–1.77 (m, 8H), 1.47–1.26 (m, 72H), 0.88 (t, J = 7.2 Hz, 12H); ^{13}C NMR (100 MHz, $CDCl_3$) δ (ppm) 164.8, 149.6, 149.3, 133.8, 127.8, 121.6, 114.7, 113.7, 69.43, 69.37, 67.3, 31.9, 29.69, 29.65, 29.6, 29.45, 29.42, 29.37, 29.34, 26.28, 26.06, 26.04, 22.7, 14.1. A part of signals

corresponding to dodecyloxy groups were overlapped; MS (APCI, positive): *m*/*z* calcd. for $C_{66}H_{112}O_8$ 1032.8352 $[M]^+$, found 1032.8379.

Synthesis of 1. To a solution of **4** (1.25 g, 1.21 mmol) in CH_2Cl_2 (30 mL) was added tri-*n*-butylphosphine (330 μL, 1.32 mmol) dropwise at 0 °C. After stirring at room temperature under a nitrogen atmosphere for 40 min, the reaction mixture was added dropwise via a syringe to a solution of **6** (209 mg, 0.50 mmol) in CH_2Cl_2 (60 mL) at 0 °C, and then 1,8-diazabicyclo[5.4.0]undec-7-ene (DBU, 5 μL, 33 μmol) was added. After stirring at 50 °C for 2 days, the reaction was quenched with water and the reaction mixture was extracted with CH_2Cl_2. The extract was washed with brine and dried over anhydrous Na_2SO_4. After removal of the solvent under reduced pressure, the residue was purified by column chromatography over silica gel using CH_2Cl_2/hexane/Et_3N (48/2/1) followed by HPLC using $CHCl_3$/Et_3N (49/1) to afford **1** (344 mg, 0.14 mmol, 28%) as a yellow solid.
^{1}H NMR (400 MHz, $CDCl_3$) δ (ppm) 8.324 (s, 4H), 8.315 (s, 4H), 7.82 (s, 4H), 7.16 (s, 4H), 6.95 (d, J = 2.0 Hz, 4H), 6.91 (dd, J = 8.0 and 2.0 Hz, 4H), 6.83 (d, J = 8.0 Hz, 4H), 5.18 (s, 8H), 4.00–3.95 (m, 16H), 1.83–1.77 (m, 16H), 1.48–1.41 (m, 16H), 1.33–1.23 (m, 128H), 0.90–0.85 (m, 24H); ^{13}C NMR (100 MHz, $CDCl_3$) δ (ppm) 167.5, 149.4, 149.2, 135.7, 133.1, 131.9, 131.3, 130.6, 128.2, 128.1, 127.8, 127.5, 121.5, 114.6, 113.7, 69.3, 67.6, 31.9, 29.7, 29.6, 29.46, 29.44, 29.35, 29.3, 26.1, 26.0, 22.7, 14.1. A part of signals corresponding to dodecyloxy groups were overlapped; MS (MALDI-TOF, positive): *m*/*z* calcd. for $C_{160}H_{236}NaO_{16}$ 2436.75 $[M{+}Na]^+$, found 2437.03.

Synthesis of 5. To a solution of maleic acid (1.02 g, 8.79 mmol) in DMF (30 mL) was added Cs_2CO_3 (5.70 g, 17.5 mmol), and the reaction mixture was stirred at room temperature for 90 min. After cooling to 0 °C, 3,4-dimethoxybenzyl bromide (4.00 g, 17.3 mmol) in DMF (45 mL) was added dropwise at the same temperature. After stirring at room temperature for 4 days, the reaction mixture was poured into water and then extracted with CH_2Cl_2. The extract was washed with brine and dried over anhydrous Na_2SO_4. After removal of the solvent under reduced pressure, the residue was purified by

column chromatography over silica gel using CH_2Cl_2/ethyl acetate (49/1) to afford **5** (860 mg, 2.07 mmol, 24%) as a white solid.

mp: 108.0–109.0 °C; ^{1}H NMR (400 MHz, $CDCl_3$) δ (ppm) 6.94 (dd, J = 8.0 and 2.0 Hz, 2H), 6.90 (s, 2H), 6.89 (d, J = 2.0 Hz, 2H), 6.85 (d, J = 8.0 Hz, 2H), 5.16 (s, 4H), 3.89 (s, 6H), 3.88 (s, 6H); ^{13}C NMR (100 MHz, $CDCl_3$) δ (ppm) 164.8, 149.4, 149.1, 133.8, 127.7, 121.5, 112.0, 111.2, 67.3, 56.0. A part of signals corresponding to methoxy groups were overlapped; MS (APCI, positive): m/z calcd. for $C_{22}H_{23}O_8$ 416.1466 $[M]^+$, found 416.1461.

Synthesis of 2. To a solution of **5** (102 mg, 0.245 mmol) in CH_2Cl_2 (3 mL) was added tri-*n*-butylphosphine (65 µL, 0.26 mmol) dropwise at 0 °C. After stirring at room temperature for 30 min under a nitrogen atmosphere, the reaction mixture was added dropwise to a solution of **6** (40.8 mg, 98 µmol) in CH_2Cl_2 (28 mL) at 0 °C, and then DBU (1 µL, 6.7 µmol) was added. After stirring at room temperature for 3 days, the reaction was quenched with water and the reaction mixture was extracted with CH_2Cl_2. The extract was washed with brine and dried over anhydrous Na_2SO_4. After removal of the solvent under reduced pressure, the residue was purified by column chromatography over silica gel using CH_2Cl_2/ethyl acetate/Et_3N (48/2/1) followed by recycling GPC using $CHCl_3$ and recrystallization in ethyl acetate to afford **2** (18.0 mg, 15 µmol, 15%) as a yellow solid.

m.p. >180 °C (decomp. under air); ^{1}H NMR (400 MHz, $CDCl_3$) δ (ppm) 8.334 (s, 4H), 8.326 (s, 4H), 7.82 (s, 4H), 7.17 (s, 4H), 6.95–6.84 (m, 8H), 6.30 (d, J = 8.8 Hz, 4H), 5.20 (s, 8H), 3.88 (s, 12H), 3.87 (s, 12H). ^{13}C NMR (100 MHz, $CDCl_3$) δ (ppm) 167.5, 149.2, 149.0, 135.8, 133.1, 131.9, 131.4, 130.6, 128.2, 128.1, 127.7, 127.6, 121.3, 112.0, 111.0, 67.5, 55.9. A part of signals corresponding to methoxy groups were overlapped; MS (MALDI-TOF, positive): m/z calcd. for $C_{72}H_{60}NaO_{16}$ 1203.38 $[M+Na]^+$, found 1203.16.

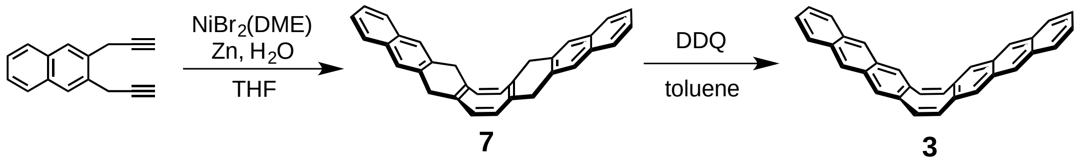

Compound **3** was synthesized through the [2 + 2 + 2 + 2] cycloaddition of a terminal diyne precursor[4] and the following DDQ oxidation. The precursor, 2,3-bis(2-propynyl)naphthalene, was synthesized according to the literature procedure[5].

Synthesis of 7. THF (9 mL) and H_2O (4.5 μL) were added to a mixture of 2,3-bis(2-propynyl)naphthalene (206 mg, 1.01 mmol), Zn (37.4 mg, 0.57 mmol), and $NiBr_2$(DME) (DME = 1,2-dimethoxyethane) (77.6 mg, 0.25 mmol), and the reaction mixture was stirred at 60 °C for 4 h under a nitrogen atmosphere. After filtration over Celite, the mixture was purified by column chromatography over silica gel using hexane/$CHCl_3$ (2/1) to afford **7** (102 mg, 0.25 mmol, 50%) as a light yellow solid.

m.p. >260 °C (decomp. under air); ^{1}H NMR (400 MHz, $CDCl_3$) δ (ppm) 7.74–7.72 (m, 4H), 7.61 (s, 4H), 7.38–7.36 (m, 4H), 5.93 (s, 4H), 3.51 (s, 8H); ^{13}C NMR (100 MHz, $CDCl_3$) δ (ppm) 133.4, 133.1, 132.7, 132.2, 127.1, 125.5, 125.2, 35.5; MS (APCI, positive): *m*/*z* calcd. for $C_{32}H_{24}$ 408.1873 $[M+H]^+$, found 408.1876.

Synthesis of 3. To a solution of **7** (275 mg, 0.67 mmol) in toluene was added 2,3-dichloro-5,6-dicyano-*p*-benzoquinone (DDQ, 386 mg, 1.70 mmol), and the reaction mixture was stirred under reflux for 2 h. After removal of the solvent under reduced pressure, the residue was washed with ethyl acetate. The residue was recrystallized from benzene to afford **3** (122 mg, 0.30 mmol, 45%) as a light yellow solid.

m.p. >300 °C (decomp. under air); ^{1}H NMR (600 MHz, $C_2D_2Cl_4$, 100 °C) δ (ppm) 8.29 (s, 4H), 7.93–7.92 (m, 4H), 7.83 (s, 4H), 7.41–7.40 (m, 4H), 7.17 (s, 4H); ^{13}C NMR (150 MHz, $C_2D_2Cl_4$, 100 °C) δ (ppm) 134.1, 132.5, 131.5, 130.1, 127.6, 127.4, 125.2, 124.8; MS (APCI, positive): *m*/*z* calcd. for $C_{32}H_{20}$ 404.1558 $[M+H]^+$, found 404.1560.

Shear experiment of the LC film of 1

Liquid crystalline nature of **1** in the mesophase (65–140 °C) was confirmed by a shear experiment (Supplementary Fig. 1). A 3-μm-thick film of **1** was prepared between glass plates. At 100 °C, the glass plate covered on the LC film was slid in a single direction with the bottom glass plate fixed. The sample was cooled to room temperature and observed by polarized optical microscopy (POM) under the crossed Nicols. When the analyzer and polarizer were both rotated by 45°, the contrast of the POM image clearly changed. Moreover, the POM image turned back to an initial state when the analyzer and polarizer were further rotated by 45° in the same direction, indicating the anisotropy of the molecular alignment in the shear direction.

XRD measurement of the bulk LC sample of 1

Compound **1** was melted at 160 °C and poured into a glass capillary under vacuum. The XRD measurement of the bulk sample using Cu Kα radiation (λ = 1.5418 Å) was performed at 100 °C in the LC phase (Supplementary Fig. 2). Miller indices (hkl) of the observed diffraction peaks were determined based on the standard peaks at $2\theta = 2.60$ and 3.98°, which was assigned as (200) and (220), respectively. Structural parameters are discussed in Methods of the main text. In consideration of the molecular size and the sample density, the number of molecules in the repeating cell unit of the liquid crystal phase was estimated to be $Z = 4$, which suggests a *side-by-side* packing of the columnar array structures as demonstrated in Supplementary Fig. 3. The XRD measurement was also performed at 25 °C in the solid phase, in which the bulk sample was prepared by cooling the liquid crystalline sample at the rate of 5 °C min^{-1} (Supplementary Fig. 4). The result suggested that the solid phase is a LC glass state, whose packing structure is comparable with that in the LC phase.

Single crystal X-ray structure analysis of 2

Structural parameters are described in Fig. 2b and Methods of the manuscript. Mo Kα radiation (λ = 0.7107 Å) was used for the measurement. The oscillation angle and camera distance were 0.5° and 75 mm, respectively. The X-ray exposure time per frame was 160 sec. Data sets of 1440 frames were integrated and scaled with the CrystalClear-SM Expert 2.0 r4 program (Rigaku, 2009). The structures were solved by direct method (SHELXS-2013) and refined by least-squares calculations on F^2 for all independent reflections (SHELXL-2013)[6]. All non-hydrogen atoms were refined with anisotropic displacement parameters, and hydrogen atoms were placed in idealized positions and refined by applying riding models with the relative isotropic displacement parameters. The deposition number of the Cambridge Crystallographic Data Centre: 1054572.

Thermography analysis after the photoirradiation on the LC film of 1

A 5-μm-thick LC film of **1** was prepared between glass plates, where the temperature was kept at 100 °C on a hot stage. The LC film turned into liquid by the UV-LED irradiation (160 mW cm^{-2}) in 10 sec. Before and after the UV irradiation, thermography analysis was conducted to confirm the negligible temperature increase (Supplementary Fig. 7).

Analyses of the photoproducts after the photoirradiation on the LC film of 1

The 1-µm-thick film of **1** between glass plates was irradiated at 100 °C with a hand-held UV lamp (365 nm, 3.2 mW cm^{-2}) for 100 sec. The resulting products were analyzed by MALDI-TOF MS and subjected to recycling GPC (Supplementary Fig. 8). The residue mainly consisted of the unreacted monomer **1** and its photodimer, although a small amount of oligomeric products were included in the isotropic mixture. The photoproducts ratio among the monomer/dimer/oligomers of **1** is dependent on the total irradiation dose as well as the film thickness. When the 365-nm UV irradiation with a total dose of 320 mJ cm^{-2} was performed on a 1-µm-thick LC film at 100°C, half of the monomer **1** was consumed for the dimerization.

The isotropic mixture after the photoirradiation was subjected to GPC, and then the photodimer of **1** (compound **8**) was isolated as a main product except for the unreacted monomer **1**. The less symmetric structure of the photodimer **8** was determined by the ^{1}H and ^{13}C NMR analyses (Supplementary Figs 9 and 10).

The thermal back reaction of the isolated photodimer **8** into **1** was also demonstrated in the ^{1}H NMR analysis. Upon heating the solution of **8** in $C_2D_2Cl_4$ at 130 °C, the monomer **1** was obtained after 9 h (Supplementary Fig. 11).

The thermal recovery of the LC film of **1** from the photoirradiated fluid mixture was supported by the POM observation (Supplementary Fig. 12).

UV-visible absorption and fluorescence spectra of the isolated photodimer **8** were measured in CH_2Cl_2 (Supplementary Fig. 13). The blue fluorescence of **8** is consistent with the result of the fluorescence color change during the photoirradiation on the LC film of **1** (Fig. 4), which elucidated the processes of the *in situ* generation of **8** and the following thermal back reaction at 160 °C (Supplementary Fig. 14).

GI-XRD measurement of the spin-coated film of 1

A spin-coated film of **1** with the thickness of *ca.* 450 nm was prepared on a quartz glass plate. However, the high-temperature GI-XRD measurement using this film failed because of a dewetting behavior during the temperature control. In order to avoid the dewetting, the spin-coated film was exposed to ozone atmosphere for 120 sec, and then a 1 wt% aqueous solution of polyvinyl alcohol (PVA) was spin-coated over the sample at 1000 rpm for 60 sec. The ozone treatment was performed to form a hydrophilic surface for coating the film with a PVA layer. This ozone treatment only leads to a surface modification rather than an internal exposure, which was confirmed by the fact that the absorption spectrum of the spin-coated film did not change upon the ozone treatment. Thus the surface modification has little influence on the following observations.

The spin-coated film was initially annealed at 160 °C for 15 min and gradually cooled to 100 °C, and then the GI-XRD measurement was conducted (Supplementary Fig. 15). Incident angles of X-ray beam to the films were set at 0.18–0.22° using pulse controllers. In this experiment, the 2D diffractions of the sample were observed as an almost angle-independent image, although a specular reflection and a weak diffuse scattering were observed. This result support that the columnar LC arrays are randomly oriented in the thin film. To avoid counting a specular reflection observed in the out-of-plane direction (ϕ = 90°), the 2D diffractions were integrated in the range of ϕ = 30–60°. After UV light irradiation at 30 mW cm^{-2} for 5 sec at the same temperature (100 °C), a change in the diffraction pattern was observed. After that, the sample was heated at 160 °C for 20 min and gradually cooled to 100 °C, and then the recovery of the diffraction pattern was confirmed. This result supported the photoinduced reaction of **1** and its thermal back reaction in the spin-coated film.

Film preparation between two glass plates

Powder sample of **1** was sandwiched between glass plates. The sample was melted by heating the glass plates at 160 °C for 15 min on a hot plate. The glass plates were gradually cooled to room temperature, affording the film of the pure compound **1** between the glass plates. The thickness was measured using a digital micrometer.

Particularly in the experiment of the shear strength measurement, 130-μm-thick films of **1** were uniformly prepared between glass plates (Supplementary Fig. 16). A 130-μm-thick PTFE silicone tape with a 6 mmϕ punched hole was placed on a glass plate as a spacer. Powder sample of **1** was placed in the hole surrounded by the PTFE silicone tape, and the sample was heated above the melting point of 140 °C. The sample was covered with the other glass plate and a 0.5 kg weight was placed on the glass plates at the same temperature. Then, the sample was cooled for 30 min to prepare a 130-μm-thick film. In this procedure, the temperature was controlled on a hot plate and monitored by an infrared thermometer.

General notes for the cohesive force and the adhesion force

In general, adhesive strength is discussed with cohesive force and adhesion force (Supplementary Fig. 17)[7]. The cohesive force F_{cohesion} derives from the internal strength of the adhesive material, which is closely related to the intermolecular interaction particularly in the case of small molecules. On the other hand, the adhesion force F_{adhesion} is the interaction between the adhesive material and the substrate surface. If $F_{\mathrm{cohesion}} > F_{\mathrm{adhesion}}$, the bonding strength is determined by the adhesion force and thus largely depends on the surface conditions of the substrates such as hydrophilicity. When $F_{\mathrm{cohesion}} < F_{\mathrm{adhesion}}$, in contrast, the bonding strength is determined by the cohesive force regardless of the surface conditions.

The exhibition mechanism of the adhesion force has also been widely studied both in molecular to macroscopic scales in relation to the rheology of the materials at interface[8].

Preparation of the spin-coated films of 1 for the light transmittance measurement

The spin-coated films of **1** were prepared with various thicknesses (See Methods in the main text). UV-visible absorption measurement of these thin films was conducted (Supplementary Fig. 18) and the transmittance at 365-nm wavelength was plotted (Fig. 4a). The film thickness was determined by the AFM analysis. After the thin film was scratched with a spatula, the difference in the height between scratched and undamaged area was measured. According to the height profile along the direction perpendicular to the scratched line, the thickness of the spin-coated film was estimated.

Light transmittance of the spin-coated films of 1

The logarithmic values of the light transmittance T (= I/I_0) at 365-nm wavelength were plotted against the corresponding film thicknesses L (Fig. 4a). In this figure, the fitting curve was delineated according to the following equation (Supplementary Equation 1). In consideration of a transmittance loss, the Lambert–Beer law was described below:

$$\log_{10}\{ I_0 (1 - i) / I \} = \varepsilon\, c\, L \qquad [1]$$

I_0 : Intensity of incident light

I : Intensity of transmitted light

ε : Molar absorption coefficient of the compound

c : Concentration of the compound

i : Ratio of a transmittance loss except for absorption

According to this equation, the absorbance $\log(I_0/I)$ is demonstrated as follows (Supplementary Equation 2):

$$\log(I_0/I) = \varepsilon\, c\, L - \log(1-i) \qquad [2]$$

The absorbance of the samples at the 365-nm wavelength showed a linear fitting against the film thickness with the coefficient of determination R^2 = 0.997 (Supplementary Fig. 19). On the basis of the fitting parameters obtained here, the light transmittance T (= I/I_0) was delineated as the exponential function of the film thickness (Fig. 4a).

Demonstration of the small adhesive residue on the UV irradiated glass plate

Relative amounts of the adhesive residues on the glass plates were compared after the photoseparation at 100 °C (Supplementary Fig. 20). First, two films of **1** with the same thickness (20 μm) were prepared. As for one sample, the 365-nm light was irradiated using a hand-held UV lamp (3.2 mW cm^{-2}) from the face of a glass plate attached to the weight until the glass plates were separated in *ca.* 100 sec (Supplementary Fig. 20a). As for the other sample, the UV irradiation was performed from the face of a hanged glass plate (Supplementary Fig. 20b). Then, the residues adhering to the dropped glass plate and to the hanged glass plate were individually dissolved in the same amount of CH_2Cl_2 (500 mL), and then the absorption spectra of these solutions were compared.

When the light was irradiated from the face of the covered glass plate, the absorbance of the residue solution obtained from the dropped glass plate was significantly smaller than that from the hanged glass substrate (Supplementary Fig. 20a). On the other hand, the tendency was reversed when the light irradiation was performed from the opposite direction (Supplementary Fig. 20b). These results demonstrated that the photoinduced melting of the adhesive film took place near the interface within a few micrometers range from the irradiated surface.

Theoretical calculations

The density functional theory (DFT) and time-dependent DFT (TD-DFT) calculations of compound **3** were performed at the PBE0/def-SV(P) and TD-PBE0/def-SV(P) levels of theory. Ground and excited state geometries for **3** have been fully optimized, and a relaxed potential energy scan was performed from the Franck-Condon geometries to the minima on the lowest excited singlet state. The energy levels of the ground state S_0 and excited states S_a, S_b, S_c, and S_d with fixed bent angle φ of the central 8-membered ring are listed in Supplementary Fig. 21. Kohn-Sham molecular orbitals of **3** and their corresponding orbital energies for different bent angles are shown in Supplementary Fig. 22. The effect of antiaromaticity of the planar 8-membered ring (cyclooctatetraene) on their orbital energies has been previously reported.[9]

Photophysical properties of 1 and 3

UV-visible absorption, fluorescence, and excitation spectra of **1** measured in various organic solvents showed the small solvent dependence (Supplementary Fig. 24). Large Stokes shift observed in solution indicated a significant conformational change of **1** in the photoexcited state (S_1), which was suppressed in the polymethyl methacrylate (PMMA) matrix (see Fig. 5b). Similar results were obtained for the main hydrocarbon framework **3** (Supplementary Fig. 23). As expected in the DFT calculations (Fig. 5a), the conformational change of **3** in S_1 should occur from the V-shaped form into the planar form, which was suppressed in the PMMA matrix as well as in the frozen media.

Supplementary References

1. Lin, C.-H., Lin, K.-H., Pal, B. & Tsou, L.-D. Iterative synthesis of acenes via homo-elongation. *Chem. Commun.* 803–805 (2009).
2. Zhang, S. & Zhao, Y. Facile preparation of organic nanoparticle by interfacial cross-linking of reverse micelles and template synthesis of subnanometer Au–Pt nanoparticles. *ACS Nano* **5**, 2637–2646 (2011).
3. Yuan C. *et al.* A π-conjugated system with flexibility and rigidity that shows environment-dependent RGB luminescence. *J. Am. Chem. Soc.* **135**, 8842−8845 (2013).
4. Wender, P. A. & Christy, J. P. Nickel(0)-catalyzed [2 + 2 + 2 + 2] cycloadditions of terminal diynes for the synthesis of substituted cyclooctatetraenes. *J. Am. Chem. Soc.* **129**, 13402–13403 (2007).
5. Taillemite, S. & Fichou, D. Synthesis of the first tetracene–[60]fullerene dyad. *Eur. J. Org. Chem.* **2004**, 4981–4984 (2004).
6. Sheldrick, G. M. Crystal structure refinement with SHELXL. *Acta Cryst.* C**71**, 3–8 (2015).
7. Silva, L. F. M., Öchsner, A. & Adams, R. D. (eds) *Handbook of adhesion technology* (Springer-Verlag, 2011).
8. Leger, L. & Creton, C. Adhesion mechanisms at soft polymer interfaces. *Phil. Trans. R. Soc. A* **366**, 1425–1442 (2008).
9. Yuan C. *et al.* Hybridization of a flexible cyclooctatetraene core and rigid aceneimide wings for multiluminescent flapping π systems. *Chem. Eur. J.* **20**, 2193−2200 (2014).
